# Comparative metabolomics study on therapeutic mechanism of electro-acupuncture and moxibustion on rats with chronic atrophic gastritis (CAG)

**DOI:** 10.1038/s41598-017-13195-5

**Published:** 2017-10-30

**Authors:** Cai-chun Liu, Jiao-long Chen, Xiao-rong Chang, Qi-da He, Jia-cheng Shen, Lin-yu Lian, Ya-dong Wang, Yuan Zhang, Fu-qiang Ma, Hui-ying Huang, Zong-bao Yang

**Affiliations:** 1 Department of Traditional Chinese Medicine and Shenzhen Research Institute, Xiamen University, Xiamen, 361005 China; 2 College of Acupuncture and Moxibustion, Fujian university of Traditional Chinese Medicine, Fuzhou, 350122 China; 3 College of Acupuncture and Moxibustion, Hunan university of Traditional Chinese Medicine, Changsha, 410208 China; 4 School of Life Sciences, Xiamen University, Xiamen, 361005 China

**Keywords:** Metabolomics, Gastrointestinal diseases

## Abstract

Some studies have proved that both acupuncture and moxibustion are very effective for the treatment of CAG. However, little is known about therapeutic mechanism of electro-acupuncture and moxibustion on CAG as well as the difference between them. On the other hand, metabolomics is a ‘top-down’ approach to understand metabolic changes of organisms caused by disease or interventions in holistic context, which consists with the holistic thinking of electro-acupuncture and moxibustion treatment. In this study, the difference of therapeutic mechanism between electro-acupuncture and moxibustion on CAG rats was investigated by a ^1^H NMR-based metabolomics analysis of multiple biological samples (serum, stomach, cerebral cortex and medulla) coupled with pathological examination and molecular biological assay. For all sample types, both electro-acupuncture and moxibustion intervention showed beneficial effects by restoring many CAG-induced metabolic changes involved in membrane metabolism, energy metabolism and function of neurotransmitters. Notably, the moxibustion played an important role in CAG treatment mainly by regulating energy metabolism in serum, while main acting site of electro-acupuncture treatment was nervous system in stomach and brain. These findings are helpful to facilitate the therapeutic mechanism elucidating of electro-acupuncture and moxibustion on CAG rats. Metabolomics is promising in mechanisms study for traditional Chinese medicine (TCM).

## Introduction

Chronic atrophic gastritis (CAG) is one of the most common functional gastrointestinal disorders and characterized by fullness, epigastric pain, belching and anorexia^1^. It is widely known that CAG is an important precursor lesion of gastric cancer, which is the second leading cause of cancer-related deaths worldwide^2,3^. In modern medicine, CAG is mainly treated by synthetic medicine such as folic acid as well as vitamin B(12)^4^, or surgery including helicobacter pylori eradication and endoscopic minimally invasive treatment^5,6^. And these treatments often bring an significant effect, while limited in clinical application because of high cost, longer course of treatment and adverse effects^7^. So, the search of a complementary and alternative intervention to treat CAG is desperately urgent. Currently, the traditional Chinese medicine (TCM) is increasingly embraced by the people at home and abroad because of their high therapeutic performance and few side-effects^8^. Therefore, an ideal strategy from TCM to relieve CAG represents urgent clinical demands.

As the major components of TCM, acupuncture and moxibustion have been used widely in clinic in China for thousands of years and are called “green therapy” because of their security, economic and few side effects^9,10^. Electro-acupuncture is an improved version of Chinese traditional acupuncture, which stimulates acupuncture points by manual manipulations of needles. Electro-acupuncture increases stimulation to improve the clinical effects by delivering electrical pulses to needles^11^. Moxibustion is conducted to stimulate acupoints by the heat from moxa burning^12^. Electro-acupuncture and moxibustion are believed to have similar clinical effects, although different in their stimulation^13^. Currently, both acupuncture and moxibustion have been shown to be effective on CAG in several studies^14,15^. And it has been reported that acupuncture can down-regulate the expression of miR-21, NF-γB p65 and miR-155 and up-regulate the expression of miR-146a in rats with CAG^16^. In our previous study, it was found that moxibustion can significantly decrease (epidermal growth factor) EGF, (transforming growth factor-α)TGF-α, (proliferating cell nuclear antigen)PCNA, (vascular endothelial growth factor)VEGF, (Ag-nucleolar organization regions)Ag-NORs in gastric mucosal of rats with CAG^17^. The differential expression of genes leads us to much better understanding of CAG as well as mechanism of acupuncture and moxibustion. But it is only part of the information we need because organisms often respond in complex and unpredictable ways to stimuli that causes disease^18^. To give a more complete picture, the metabolic differences of biological status we need to observe.

Metabolomics is a powerful system biological approach, which can simultaneously monitor and evaluate changes of global metabolic compositions involved in biochemical processes caused by changes in disease and other stimulations in a holistic context^19^. This specific feature is concordant with the holistic view of TCM^20^. NMR is one of most preferred platforms and extensively used in metabolomics studies because of non-destructive and minimal sample preparation as well as nonselective analysis^21^. Recently, the application of the ^1^H NMR-based metabolomics has shown value in elucidation of the therapeutic mechanisms of acupuncture but moxibustion is less studied^22,23^. In our preliminary study, ^1^H NMR-based metabolomics coupled molecular biology is used to analyze biological samples (stomach, cortex urine, serum and medulla) in rats with CAG and after electro-acupuncture treatment. And it was found that electro-acupuncture treatment could relieve CAG by regulating membrane catabolism, function of neurotransmitter in brain and gut microbiota metabolism^24,25^. In this study, the difference of therapeutic mechanism between electro-acupuncture and moxibustion on rats with CAG is investigated by ^1^H NMR-based metabolomics combined with pathological evaluation and molecular biological assay.

## Results

### Histological Morphology Examinations

**Figure 1:**
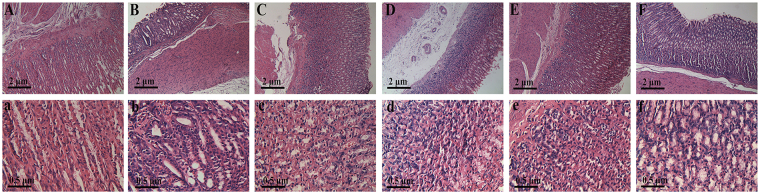
Histological examination of gastric mucosa from six groups. (**A** and a, the control rats; **B** and b, the CAG rats; **C** and c, CAG rats with electro-acupuncture treatment on the stomach meridian acupoints; **D** and d, CAG rats with electro-acupuncture treatment on the stomach meridian acupoints; **E** and e, CAG rats with moxibustion treatment on the stomach meridian acupoints; **F** and f, CAG rats with moxibustion on non-acupoints). Scale bars represent 2 μm for the top row and 0.5 μm for the bottom row.

Histological examinations of gastric tissues in rats from six groups were conducted (Fig. 1). The results of pathological examination in the control group showed that epithelial cells and glands arranged in the same size. Additionally, epithelial cells were single-layered columnar with membrane integrity and cytoplasm transparent and there were also no hyperemia and edema in the gland and mucosa (Fig. 1A and a). On the contrary, in the CAG group, reduced gland cells and thinning gastric mucosa layer were clearly seen. And cells arranged intricately with irregular nuclei and distinct nucleoli **(**Fig. 1B and b). Based on these observations, the rat modeling of CAG was a successful replication. The gastric mucosas of CAG rats with electro-acupuncture or moxibustion treatment on the stomach meridian acupoints were improved in different degrees with moderate thickness of gastric mucosa and regular gland arrangement (Fig. 1C,c,E and e). These observations illustrate a significant curative effect of electro-acupuncture and moxibustion on rats with CAG. In the other hand, thinning gastric mucosa layer without obvious improvement, incomplete mucosa gland morphology and irregular cell alignment were shown in CAG rats with electro-acupuncture or moxibustion on non-acupoints (Fig. 1D,d,F and f). It suggests that electro-acupuncture and moxibustion treatment on non-acupoints has no much effect on rats with CAG.

### Brain-gut Peptide (BGP) Examinations by ELISA

**Figure 2:**
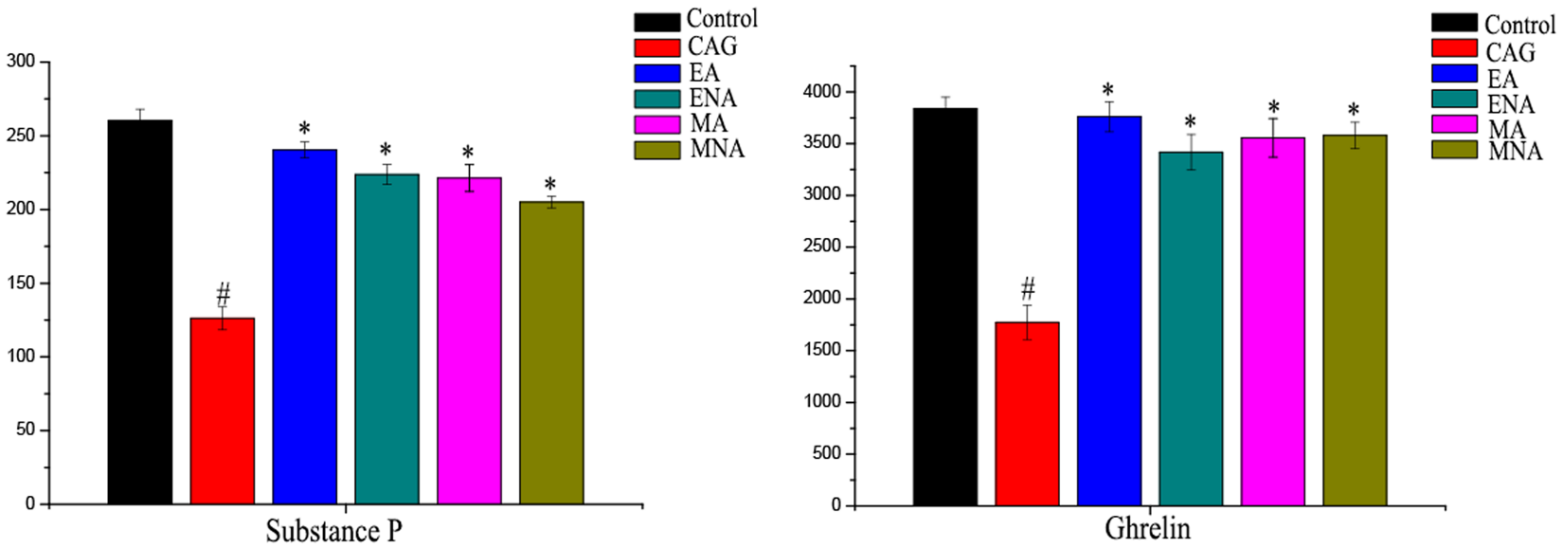
The expression of substance P and ghrelin in gastric mucosa of rats in six groups. (Control, control rats; CAG, chronic atrophic gastritis rats; EA, CAG rats with electro-acupuncture treatment on the stomach meridian acupoints; ENA group, CAG rats with electro-acupuncture on non-acupoints; MA group, CAG rats with moxibustion treatment on the stomach meridian acupoints; MNA group, CAG rats with moxibustion on non-acupoints). (^#^Means a statistical significance p < 0.05 when compared with the control group; *means a statistical significance p < 0.05 when compared with the CAG group.)

In this study, the levels of substance P and ghrelin in gastric mucosa of rats were determined by ELISA. And it was found that the low expression levels of the substance P and ghrelin in gastric mucosa of rats in CAG group compared with the controls. In the other hand, the levels of these two factors in gastric mucosa and intestinal mucosa of CAG rats with electro-acupuncture or moxibustion treatment on the stomach meridian acupoints were increased to the normal (Fig. 2).

### ^1^H NMR Profiles of Serum and Tissues from Rats

**Figure 3:**
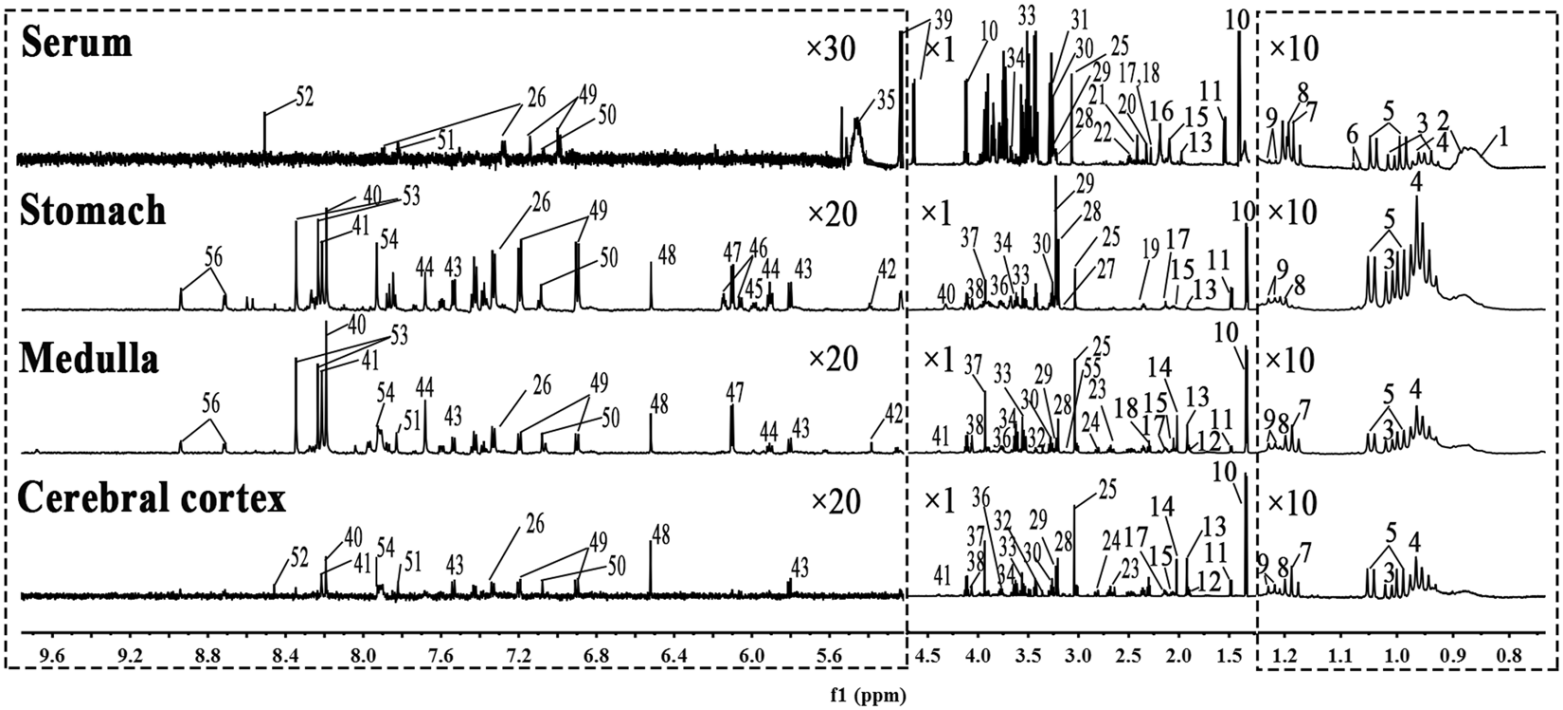
Typical ^1^H NMR spectra of serum and aqueous extracts from stomach, medulla and cerebral cortex tissues. (1, Low density lipoprotein; 2, Very low density lipoprotein; 3, Isoleucine; 4, Leucine; 5, Valine; 6, 2-Ketobutyric acid; 7, Ethanol; 8, β-OH-butyrate; 9, Methylmalonate; 10, Lactate; 11, Alanine; 12, γ-Aminobutyrate; 13, Acetate; 14, N-acetyl aspartate;15, Glutamate; 16, O-acetyl glycoprotein; 17, Glutamine; 18, Methionine; 19, Glutathione; 20, Acetone; 21, Acetoacetate; 22, Citrate; 23, Aspartate; 24, N,N-dimethylglycine; 25, Creatinine; 26, Phenylalanine; 27, Ethanolamine; 28, Choline; 29, Phosphocholine; 30, Glycerophosphocholine; 31, Betaine; 32, Inositol; 33, Glycine; 34, Glycerol; 35, Glycogen; 36, Serine; 37, Phosphocreatine; 38, Adenosine monophosphate; 39, Glucose; 40, Hypoxanthine; 41, Inosine; 42, Allantoin; 43, Uracil; 44, Uridine; 45, Uridine diphosphate glucose; 46, NADP+; 47, Inosinic acid; 48, Fumarate; 49, Tyrosine; 50, Histidine; 51, Methylhistidine; 52, Formate; 53, Adenosine; 54, Xanthine; 55, Malonic acid; 56, Nicotinamide).

The typical ^1^H NMR spectra of serum and extracts from tissues (stomach, cerebral cortex and medulla) are shown in Fig. 3. Additionally, two-dimensional (2D) NMR spectra including ^1^H- ^1^H correlation spectroscopy (COSY) and ^1^H- ^13^C heteronuclear single quantum coherence (HSQC) for the samples were also acquired (Figs S2–9). And the main metabolites from the spectra were identified according to published references, our own developed NMR database and Human Metabolome Database (HMDB: http://www.hmdb.ca/) (in Tables S1–4).

**Figure 4:**
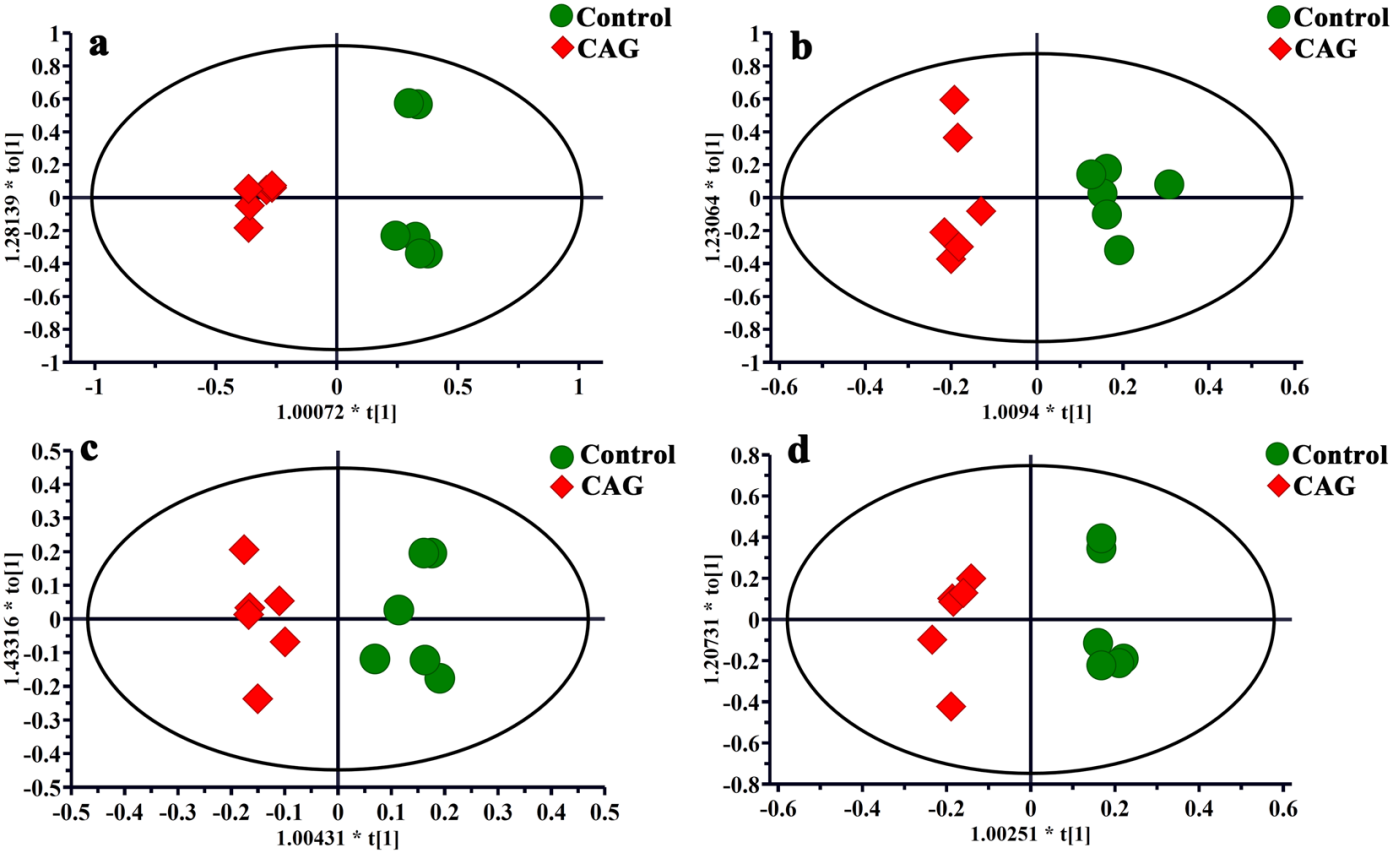
OPLS-DA scores plots from the CAG rats and the controls in serum (**a**, R2X = 0.741, R2Y = 0.982, Q2 (cum) = 0.889), stomach (**b**, R2X = 0.552, R2Y = 0.943, Q2 (cum) = 0.487), medulla (**c**, R2X = 0.69, R2Y = 0.942, Q2 (cum) = 0.599) and cerebral cortex (**d**, R2X = 0.665, R2Y = 0.98, Q2 (cum) = 0.726) tissues. For all sample types, a good separation between the control group and CAG group was initially observed, indicating a noticeable change in metabolic profile occurred in CAG group.

Visual inspection of these ^1^H NMR spectra exhibited less obvious difference among these groups because of the complexity of the spectra. So, all of resulting datasets were subsequently analyzed to examine the clustering of each group by OPLS-DA, which can reveal any possible variables contributing to CAG molding and electro-acupuncture or moxibustion treatment. For all sample types, a good separation between the control group and CAG group was initially observed in Fig. 4, indicating a noticeable change in metabolic profile occurred in CAG group. We also performed the corresponding S-plot and t-test for metabolites related CAG modeling (in supplementary Fig. S10), which showed some changes in levels of metabolites occurred in CAG group compared with the controls as follows: a)the serum levels of glycogen, glucose and acetoacetate were increased, whereas the levels of methionine, lactate and betaine were decreased; b) the levels of glutathione and glutamine were up-regulated, while the level of ethanolamine was down-regulated in stomach tissue; c) the higher levels of inosine, methylmalonate and malonic acid occurred but lower levels of phenylalanine and inositol were presented in medulla tissue; d) the increased levels of hypoxanthine, nicotinamide and glycerol occurred in cerebral cortex tissue.

**Figure 5:**
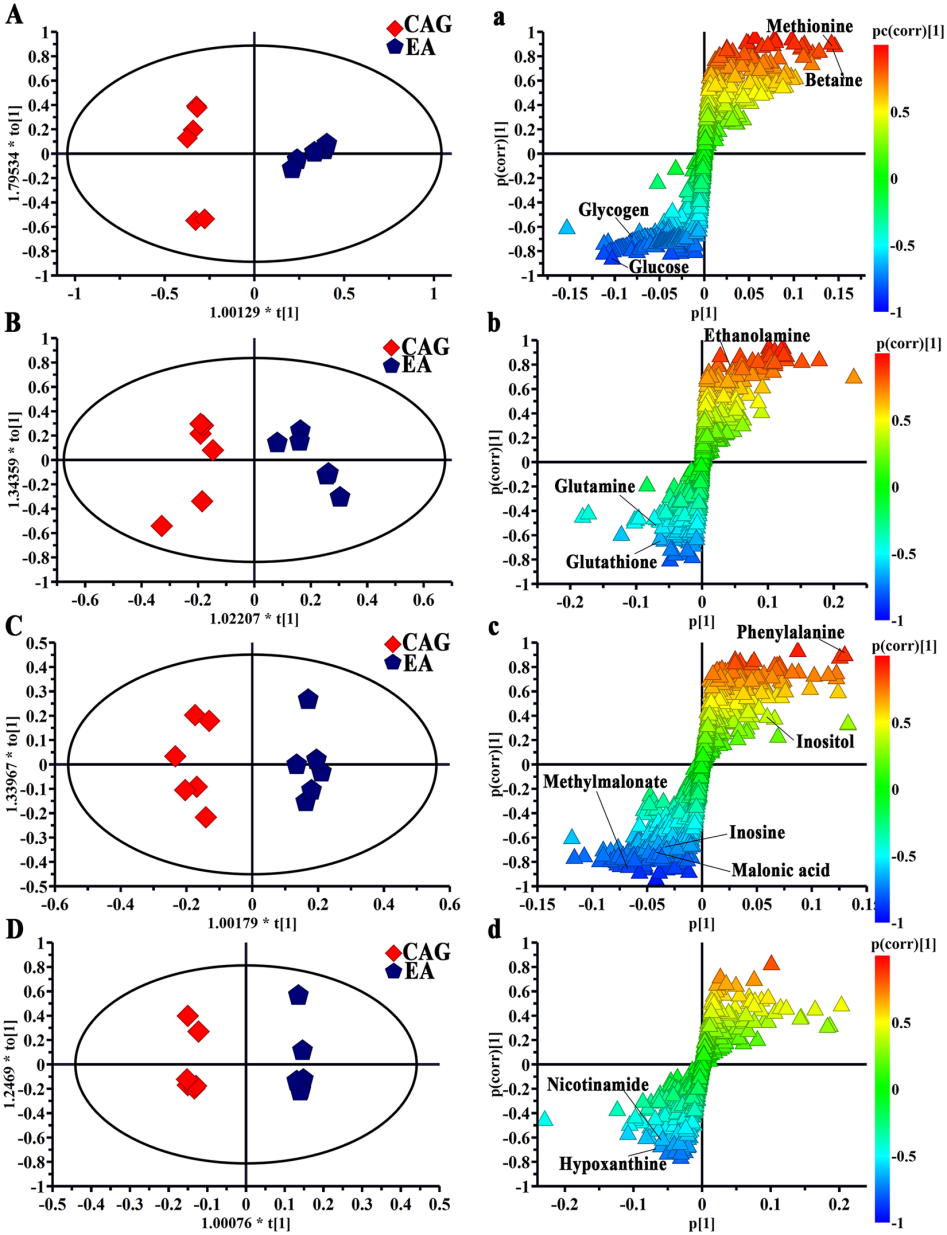
OPLS-DA scores plots and corresponding S-plots from the CAG rats and the CAG rats with electro-acupuncture treatment on the stomach meridian acupoints in serum (**A** and a, R2X = 0.787, R2Y = 0.97, Q2 (cum) = 0.805), stomach (**B** and b, R2X = 0.561, R2Y = 0.901, Q2 (cum) = 0.764), medulla (**C** and c, R2X = 0.587, R2Y = 0.972, Q2 (cum) = 0.571) and cerebral cortex (**D** and d, R2X = 0.738, R2Y = 0.995, Q2 (cum) = 0.5) tissues) tissues.

**Figure 6:**
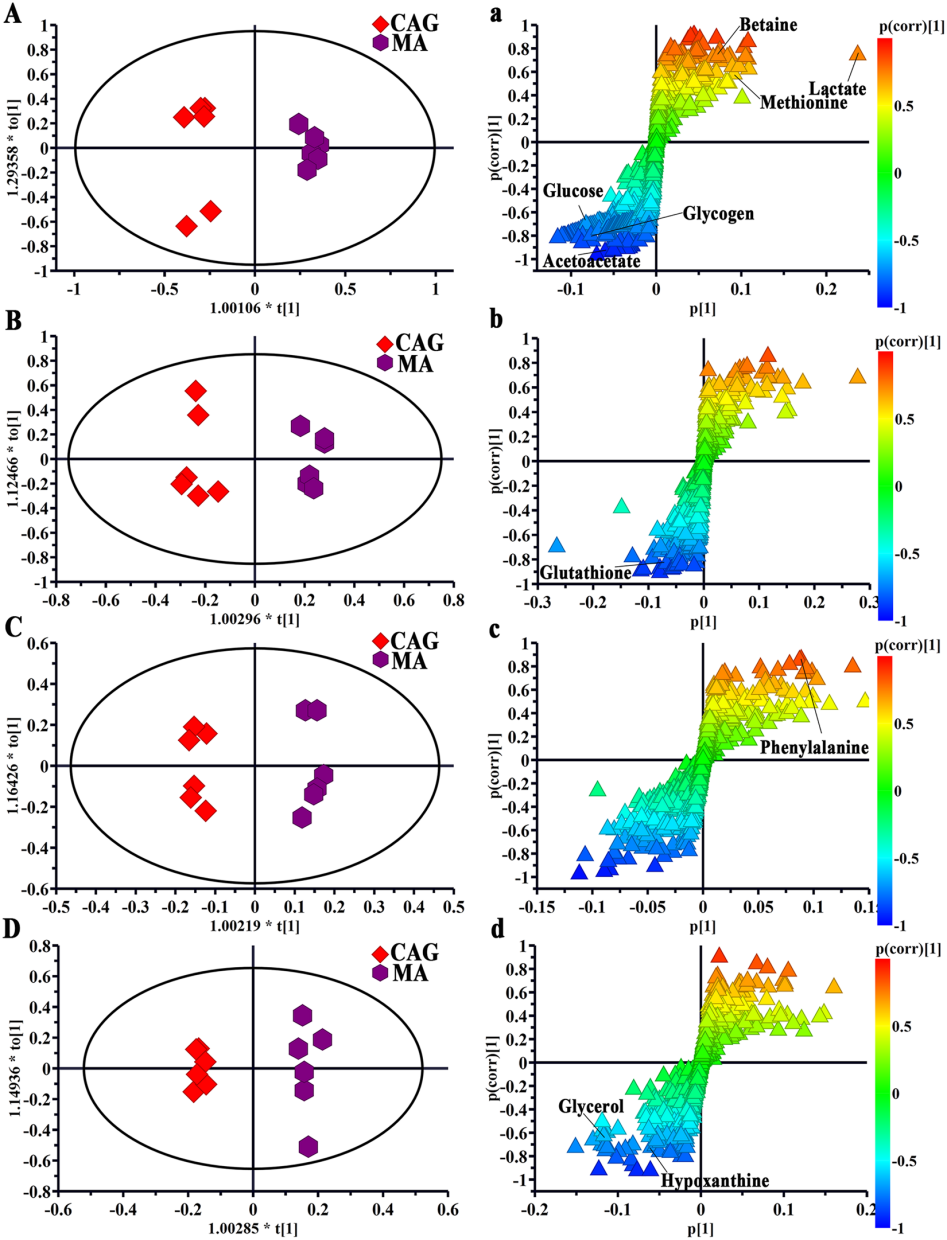
OPLS-DA scores plots and corresponding S-plots from the CAG rats and the CAG rats with moxibustion treatment on the stomach meridian acupoints in serum (**A** and a, R2X = 0.845, R2Y = 0.978, Q2 (cum) = 0.908), stomach (**B** and b, R2X = 0.656, R2Y = 0.97, Q2 (cum) = 0.822), medulla (**C** and c, R2X = 0.586, R2Y = 0.985, Q2 (cum) = 0.762)and cerebral cortex (**D** and d, R2X = 0.554, R2Y = 0.987, Q2 (cum) = 0.789) tissues.

Using the strategy mentioned before, all of rats with electro-acupuncture or moxibustion treated presented a clear separation from CAG rats, illustrating that both the electro-acupuncture and moxibustion had a good effect on CAG (in Figs 5 and 6). And the corresponding S-plots and the following t-test suggested as follows: a) levels of glycogen, methionine, betaine, glucose, glutathione, phenylalanine and hypoxanthine were reversed by both electro-acupuncture and moxibustion treatment; b) the levels of glutamine, ethanolamine methylmalonate, malonic acid, inositol, inosine and nicotinamide were returned to normal levels after electro-acupuncture treatment while lactate, acetoacetate and glycerol were regulated by moxibustion treatment.

### Correlation analysis of metabolites and BGPs

The correlation analysis on the relationships between BCPs and potential metabolites from all sample types was shown in the supplementary Fig. S12. Red bars correspond to positive correlations and blue bars correspond to negative correlations. Bar length reflects the magnitude of the correlation coefficients. The bar plots analysis was performed to gain a possible relationship between metabolites and BGPs. In Fig. S12A, betaine, lactate, ethanolamine, phenylalanine and methionine were positively associated with substance P, while malonic acid, glutathione, nicotinamide, glycogen, glucose, acetoacetate, hypoxanthine, glutamine and inosine were negatively associated with substance P. In Fig. S12B, phenylalanine, methionine, betaine, inositol and lactate were positively associated with ghrelin, whereas inosine, malonic acid, acetoacetate, glycerol, glucose, nicotinamide, methylmalonate, hypoxanthine and glutamine were negatively associated with ghrelin.

## Discussion

It is known that functional dyspepsia is closely related to brain-gut peptide (BGP), which is effective in regulating various physiological functions in gastrointestinal tract by both enteric nervous system and central nervous system^26^. Substance P is a member of the BGPs and associated with the generation and signals transmission of gastrointestinal visceral hypersensitivity^27^. Ghrelin is also one of important BGPs closely related to appetite and can control gastric motility^28^. In this study, these two factors were reversed to normal by both electro-acupuncture and moxibustion treatment, indicating that both electro-acupuncture and moxibustion treatment play an important role in CAG by regulating enteric nervous system and central nervous system.

**Figure 7:**
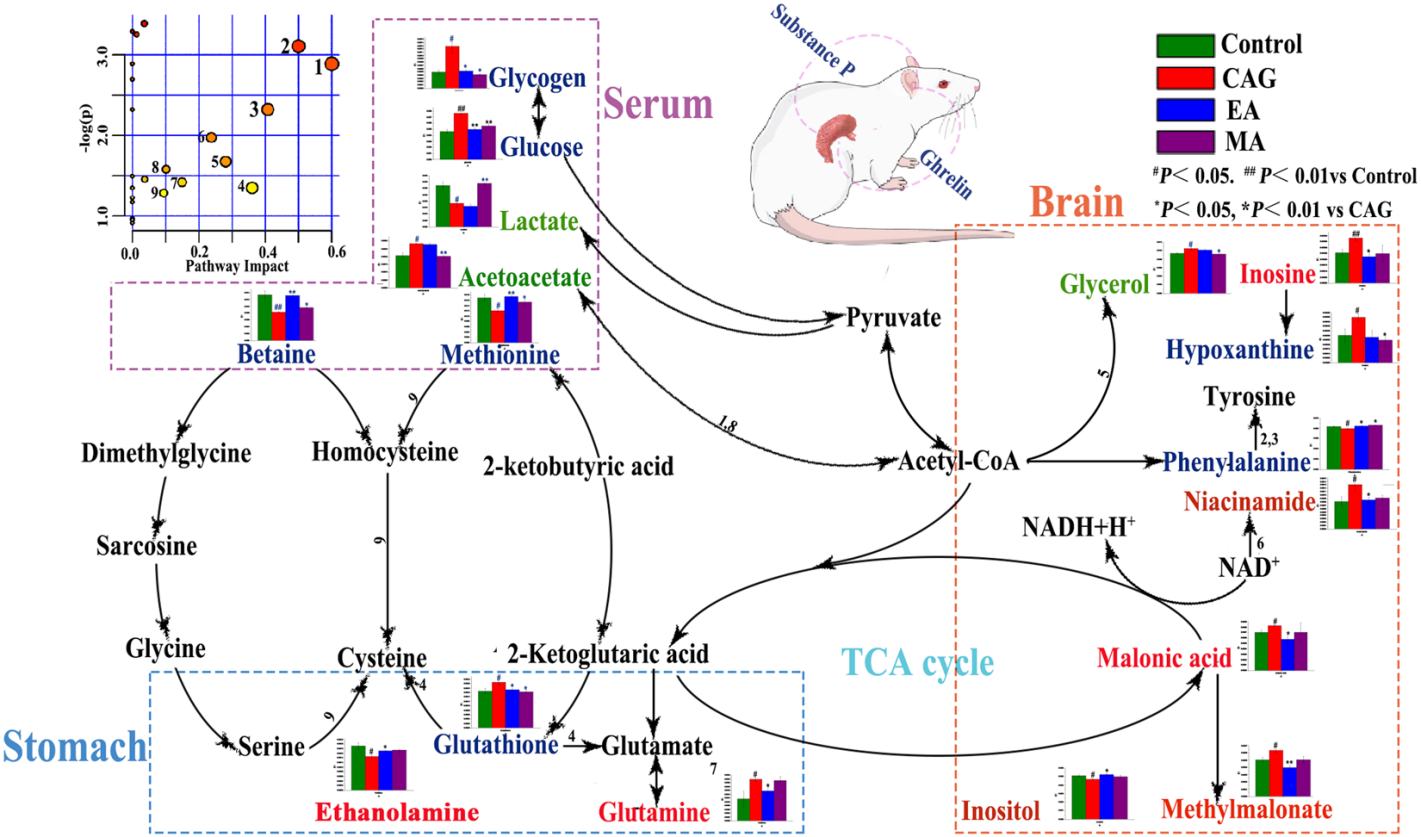
Disturbed metabolic pathways related to electro-acupuncture and moxibustion treatment on CAG. Metabolites in red, green and blue represent metabolites regulated by electro-acupuncture, moxibustion and both them, respectively. Both electro-acupuncture and moxibustion intervention showed beneficial effects by restoring many CAG-induced metabolic changes involved in membrane metabolism, energy metabolism and function of neurotransmitters. Notably, the moxibustion played an important role in CAG treatment mainly by regulating energy metabolism in serum, while main acting site of electro-acupuncture treatment is nervous system in stomach and brain. (1, Synthesis and degradation of ketone bodies; 2, Phenylalanine, tyrosine and tryptophan biosynthesis; 3, Phenylalanine metabolism; 4, Glutathione metabolism; 5, Glycerolipid metabolism; 6, Nicotinate and nicotinamide metabolism; 7, Alanine, aspartate and glutamate metabolism; 8, Butanoate metabolism; 9, Cysteine and methionine metabolism).

In the other hand, for all sample types, the different levels of changes induced by CAG-modeling were reversed by electro-acupuncture or moxibustion treatment. These results may give rise to characteristic metabolomics profiles of CAG modeling and the difference between electro-acupuncture and moxibustion treatment, which will be discussed in further detail below (Fig. 7).

### Metabolic Alterations in Serum

Serum is easy to obtain non-invasively and can provide a holistic view of the whole systems biology. Therefore, the metabolomics approach in serum is often used for disease diagnosis and drug therapy monitoring. In this study, therapeutic mechanism between electro-acupuncture and moxibustion on CAG rats were compared by a serum ^1^H NMR-based metabolomics.

Glycogen is a highly-branched polymer of about 30,000 glucose residues and principal storage form of glucose in animal and human cells. Glucose is synthesized from intermediates such as pyruvate and glycerol by a process known as gluconeogenesis and often degrades by glycolysis. And in the process of glycolysis, metabolic energy is captured^8^. Lactate is the end-product of degradation of glucose under anaerobic conditions and can be considered for the assessment of the severity of the supply/demand imbalance^29^. In the other hand, acetoacetate is one of important intermediate for synthesis and degradation of ketone bodies, which can provide acetoacetyl-CoA and acetyl-CoA for glycolysis or gluconeogenesis^30^. Functioning as a methyl donor, betaine often carries and donates methyl functional groups to facilitate necessary chemical processes such as the synthesis of dimethylglycine^31^. Particularly, it methylates homocysteine to methionine, which is considered as the metabolic precursor for cysteine^32,33^. Additionally, cysteine is important in energy metabolism^34^. So, the levels of glucose, glycogen, betaine and methionine in CAG rats were return to normal after either electro-acupuncture or moxibustion treatment, suggesting that regulating energy metabolism is important in both electro-acupuncture and moxibustion treatment on CAG rats. Only moxibustion treatment can regulate the levels of lactate and acetoacetate CAG rats, indicating that moxibustion may play a more important role in regulating energy metabolism than electro-acupuncture treatment on CAG.

### Metabolic Alterations in Extracted Stomach Tissues

Stomach was accepted as a CAG-targeted organ. As mentioned above, status of gastric mucosa of CAG rats had a different change in both histopathology and BGPs. Herein, a ^1^H NMR-based metabolomics was used to compare the therapeutic mechanism between electro-acupuncture and moxibustion on CAG rats.

Ethanolamine is a component of lecithin, which is an important part of cell membranes. Glutathione, synthesized from cysteine, glutamate and glycine, is a coenzyme in various enzymatic reactions^35^. The most important of these reactions are redox reactions, where the thiol grouping on the cysteine portion of cell membranes can protect against peroxidation^36^. In the other hand, glutathione can be converted to glutamic acid, which is a key molecule in cellular metabolism and the most abundant fast excitatory neurotransmitter in the nervous system^37^. Generally, glutamate and glutamine are inter-converted between neurons and astrocytes. In this study, the level of glutathione was reversed to normal by either electro-acupuncture or moxibustion treatment, indicating both electro-acupuncture and moxibustion treatment show beneficial effects in membrane metabolism of CAG rats. In addition, the levels of ethanolamine and glutamine could be regulate by only electro-acupuncture treatment, indicating that electro-acupuncture treatment on CAG rats may play an more important role in membrane metabolism than moxibustion treatment and can regulate neurotransmitter in nervous system.

### Metabolic Alterations in Extracted Brain Tissues

It is accepted that there is a dense neuronal network linking with stomach and central nervous system, where numerous neurotransmitters and peptides play an important role in contributing to gastric mucosal integrity^38,39^. In this study, metabolic alterations in cortex and medulla were investigated by technique of ^1^H NMR.

Malonic acid is also one of vital intermediates in the tricaboxylic acid (TCA) cycle and its derivative called methylmalonic acid can lead a block in the enzymatic conversion of methylmalonyl coenzyme A (CoA) to succinyl CoA, which plays a major role in the metabolism of fat and protein^40^. Glycerol is a three-carbon substance that forms the backbone of fatty acids in fats. When the body uses stored fat as a source of energy, glycerol and fatty acids are released. And it is not surprising that glycerol can be converted to glucose and provide energy for cellular metabolism^41^. In addition, niacinamide functions as a component of the coenzyme (nicotinamide adenine dinucleotide) NAD, which is used extensively in glycolysis and the TCA cycle of cellular respiration^42^. So, being reversed to normal in levels of malonic acid, methylmalonic acid, niacinamide by electro-acupuncture treatment is another indication about both electro-acupuncture and moxibustion treatment on CAG by regulating energy metabolism. And the level of glycerol was regulated by only moxibustion treatment, suggesting moxibustion treatment may be more important in regulating energy metabolism than electro-acupuncture treatment.

Phenylalanine is an essential amino acid highly concentrated in brain and precursor for the neurotransmitter called catecholamines^43^. Adenosine, composed of adenine and d-ribose, plays a role in signal transduction as a neurotransmitter^44^. Inosine is a purine nucleoside that is an intermediate in the degradation of purines and purine nucleosides like hypoxanthine and also occurs in the anticodon of certain transfer RNA molecules. And adenosine to inosine (A-to-I) RNA editing plays a vital role in neurotransmission in brain^45^. As a second messenger in a cell, inositol plays an important role in the form of inositol phosphates, which are important in signal transduction. In current study, the levels of these four metabolites were altered to normal by electro-acupuncture treatment while moxibustion treatment could regulate only two of them, which suggests that electro-acupuncture treatment on CAG plays a more important role in regulating nervous system than moxibustion treatment.

Together, these findings suggests that both electro-acupuncture and moxibustion treatment on CAG rats are related to regulating membrane metabolism, energy metabolism and function of neurotransmitter. From the global point of view, the main acting site of moxibustion treatment on CAG rats is blood by regulating energy metabolism, whereas electro-acupuncture treatment can ameliorate CAG mainly by repairing nervous system in stomach and brain. This is consistent with previous study about the warming effect of moxibustion can promote gastrointestinal blood circulation and enhance gastrointestinal motility energy while that clinical efficacy of acupuncture relies on the mediation of the central nervous system^46,47^.

In conclusion, a ^1^H NMR-based metabolomics analysis of multiple biological samples (serum, tomach, cortex and medulla) coupled with pathological examination and BGPs examination was used to investigate the difference of therapeutic mechanism between electro-acupuncture and moxibustion on CAG rats in this study. It was found that for all sample types in rats of CAG group, a noticeable change in metabolic profile occurred, involved in membrane metabolism, energy metabolism and function of neurotransmitter. In the other hand, both electro-acupuncture and moxibustion intervention showed beneficial effects by restoring many CAG-induced metabolic changes. Notably, the moxibustion played an important role in CAG treatment mainly by regulating energy metabolism in serum, while main acting site of electro-acupuncture treatment is nervous system in stomach and brain of CAG rats. Further studies on electro-acupuncture and moxibustion interventions by gas chromatography-mass spectrometry (GC-MS) and liquid chromatography-mass spectrometry (LC-MS)-based metabolomics are also required to validate our findings. The current work will be beneficial for better understanding difference of biological mechanism between electro-acupuncture and moxibustion treatment on CAG.

## Methods

### Ethical Statement

Animal care and experimental procedures used in this study were approved by the Animal Care and Use Committee of Xiamen University (Permit Number: SCXK 2014-0001). The study was performed in accordance with National Institutes of Health for the Care and Use of Laboratory Animals.

### Animals

36 healthy male S.D. rats (170–210 g weight) used in this study were maintained in a temperature-controlled environment (22 ± 1 °C) and a 12: 12 h light/dark cycle (lights on at 8:00 a.m.) with free access to water and food.

After adaptation for one week, all animals were randomly divided into six groups (n = 6 per group): (1) control group, (2) CAG rats, (3) CAG rats with electro-acupuncture treatment on the stomach meridian acupoints, (4) CAG rats with electro-acupuncture on non-acupoints, (5) CAG rats with moxibustion treatment on the stomach meridian acupoints and (6) CAG rats with moxibustion on non-acupoints. The rats in control group were freely accessed to water clean water and standard rat diet. According to our previous study^25^, all of rats except ones in control group were subjected to CAG modeling including compulsive sporting, irregular fasting as well as mixture of 30% alcohol and 2% sodium salicylate poured down the throats for 12 weeks.

### Electro-acupuncture and moxibustion treatment

In this study, two acupoints in the stomach meridian including Liangmen (ST 21, located 5 mm horizontally to the spot above 2cm from navel) and Zusanli (ST 36, located 5 mm below the fibular head and lateral to the anterior tubercle of the tibia) were selected according to “The Veterinary Acupuncture of China” and Government Channel and Points Standard GB12346-90 of China (Fig. S13). According to previous studies^25^, for rats in the electro-acupuncture treatment group, Liangmen (ST 21) and Zusanli (ST 36) were treated with electro-acupuncture (a 30-minute session per day) for 2 weeks after CAG modeling. While for CAG rats with electro-acupuncture on non-acupoints, the corresponding non-acupoints located 5 mm away from each of the two acupoints were treated with electro-acupuncture as mentioned above (Fig. S3). And these non-acupoints have nothing to do with the stomach meridian and do not lie on any other known acupoints. In the other hand, for CAG rats with moxibustion on acupoints and non-acupoints, the above-mentioned acupoints and non-acupoints were selected respectively and subjected to moxibustion (a 30-minute session per day) for two weeks after CAG modeling.

An electro-acupuncture apparatus (Model G6805-2; Qingdao Xinsheng Medical Instrument Factory, Shandong, China) coupled with stainless-steel acupuncture needles (0.30×40 mm) was used for two-channel electrical stimulations at irregular waves (intermittent wave: 4 Hz; irregular wave: 50 Hz) with voltage (2~4 V). For moxibustion treatment, moxa cone (height: 16 mm, diameter: 18 mm, “Han Medicine”, Nanyang, China) was placed on top of selected spots. And moxa-burning sticks were fixed to ensure that lit ends were 2 cm away from the skin.

### Histopathology

The gastric mucosa samples in rats were collected and immersed in the phosphate-buffered 10% formalin. After sample dehydration, the biopsies were embedded in wax and then sectioned at 5μm. At last, a histopathological examination by light microscopy was conducted after being stained with hematoxylin and eosin.

### Enzyme linked immunosorbent assay (ELISA) assessment

After two weeks of electro-acupuncture or moxibustion treatment, all rats were anesthetized and the gastric glands (1 cm ×1 cm) were collected. After being cut into pieces, each tissue sample was mixed with ice-cooled phosphate buffered saline (PBS) (0.1 M, 1:9 (w/v)). And then the mixture was transferred to 2 mL tube and centrifuged for 15 min (3000 rpm, 4°C) after being homogenated at 10 000 rpm on ice. Finally, the upper supernatants were collected in clean tubes and stored at −80 °C prior to analysis.

The levels of substance P and ghrelin were determined using an ELISA kit purchased from Cusabio (Wuhan, Hubei, China). The ELISA-based method was conducted according to protocol provided by manufacturer.

### Biological sample collection and ^1^H NMR experiments

After the experimental procedure, all animals were sacrificed anesthetized with isoflurane. Then the abdominal aortic blood and tissue samples were collected. The serum samples were obtained by centrifuging at 10000 rpm for 10 min at 4 °C after standing for 1h at room temperature. The cerebral cortex, medulla and stomach tissue samples were excised and snap-frozen in liquid nitrogen immediately for tissue extraction. All of samples were stored at −80 °C prior to analysis.

According to our preliminary study^25^, each serum sample of 400 μL was added to 200 μL phosphate buffer solution (90 mM K_2_HPO_4_/NaH_2_PO_4_, pH 7.4, 99.9% D_2_O) and centrifuged at 10 000 rpm for 10 min at 4 °C. And then 500 μL of supernatant was transferred into 5 mm NMR tube for NMR experiment. The ^1^H NMR spectra of serum samples were obtained by a 600 MHz Bruker spectrometer at 298 K. A standard one-dimensional (1D) Carr-Purcell-Merboom-Gill (CPMG, RD-90 (τcp-180-τcp)-acquisition) with water suppression was used to captured ^1^H NMR spectra. For every sample, 64 FIDs were collected into 20K data points over a spectral width of 12 000 Hz with a relaxation delay of 0.23 ms.

Each of pre-weighed stomach, cerebral cortex, medulla samples (200 mg) were homogenated in CH_3_OH (600 mL) and H_2_O (300 mL) and then vortexed for 1min. The samples were centrifuged for 10 min (10000 rpm, 4 °C) after partitioning on ice for 10 min. And then the upper supernatant from each sample were lyophilized and mixed with 600 μL D_2_O containing sodium 3-trimethylsilyl-(2, 2, 3, 3-d4)-1-propionate (TSP, 1 mM). Finally, a 500 μL of mixture was piped into 5 mm NMR tube for NMR analysis. ^1^H NMR spectra of these samples were also obtained using a Bruker 600 MHz spectrometer at 298 K. Standard 1D ^1^H spectra were captured with a Nuclear Overhauser Effect Spectroscopy (NOESY, RD-901-t1-90°-tm-90° -acquire) pulse sequence. For all sample, 64 FIDs were collected into 64K data points over a spectral width of 12 000 Hz with a relaxation delay of 6.5 μs.

For good metabolite identification, two-dimensional (2D) NMR spectra for the samples were also obtained by spectrometer mentioned above, including ^1^H-^1^H correlation spectroscopy (COSY) and ^1^H-^13^C heteronuclear single quantum coherence (HSQC) as our previous study^48^. The COSY spectra were acquired with a relaxation delay of 1.5s, and 48 transients were collected into 1024 data points with a spectral width of 12 ppm for both dimensions. For the 2D ^1^H-^13^C HSQC spectra, a 1.5 s relaxation delay was used for water suppression by presaturation. For each 2D spectrum, 2048×128 data points were collected using 8 scans per increment. The spectral widths were set to 12 and 240 ppm in the proton and carbon dimensions, respectively

The metabolites in NMR spectra from all samples were assigned according to published literature^21,24,25^, our own developed NMR database and HMDB database (http://www.hm db.ca/).

### Data Preprocessing and multivariate statistical analysis

The all of ^1^H NMR spectra from samples were phased and baseline corrected using MestReNova v9.0.1 software (Mestrelab Research, Santiago de Compostella, Spain). The ^1^H NMR spectra were referenced to a double peak of CH_3_ from the internal lactate at 1.33 ppm (for serum samples) or a single peak from TSP at 0.0 ppm (for tissue samples). All spectra were also peak-aligned to overcome peak-shift problem. After the chemical shift range of δ 4.70–5.2 ppm were removed to eliminate the influence of water, the spectra were segmented at δ 0.01 intervals across the region of 0.5 to 8.5 ppm(for serum samples) and 0.5–9.0 ppm (for tissue samples). To compensate for significant concentration differences between the samples, the integral values from each spectrum were normalized to a sum of all of integrals in a spectrum, and then the date matrices were formed for further multivariate analysis.

The ^1^H NMR spectral data was imported into SIMCA-P14.1 (Umetrics, Sweden) for multivariate analysis. Prior to multivariate analysis, the Pareto-scaling was used to decrease the effect of noise and artefacts in the models. Firstly, a principal component analysis (PCA) was used for a natural separation among the all groups by visual inspection of the score plots in this study. And then a supervised orthogonal projection to latent structures discriminant analysis (OPLS-DA) was carried out to distinguish different groups. Additionally, parameters for model fitness (R2) and predictive ability (Q2) were used to assess quality of OPLS-DA model. And the p-value from cross-validated analysis of variance (CV-ANOVA) was also calculated to indicate the level of significance for group separation in OPLS-DA. To discover the potential variables for differentiation, the corresponding S-plot of OPLS-DA model was conducted. The potential biomarkers were extracted according to the variable importance in the project (VIP) of the established OPLS-DA model (VIP≥1.00) and an independent-sample t-test (p <0.05) using (OriginProver.8.1). In addition, a systematically statistical correlation analysis was used to investigate the relationships between these metabolites and BCPs with online data tool Metaboanalyst 3.0 (http://www.Metaboana lyst.ca).

## Electronic supplementary material

Supplementary Information

## References

[1] Zhang Y (2016). Exploratory Factor Analysis for Validating Traditional Chinese Syndrome Patterns of Chronic Atrophic Gastritis. Evidence-based Complementary and Alternative Medicine: eCAM.

[2] Adamu MA, Weck MN, Rothenbacher D, Brenner H (2011). Incidence and risk factors for the development of chronic atrophic gastritis: Five year follow-up of a population-based cohort study. International journal of cancer.

[3] Rugge M (2016). Chronicles of a cancer foretold: 35 years of gastric cancer risk assessment. Gut.

[4] Lewerin C, Jacobsson S, Lindstedt G, NilssonEhle H (2008). Serum biomarkers for atrophic gastritis and antibodies against Helicobacter pylori in the elderly: Implications for vitamin B12, folic acid and iron status and response to oral vitamin therapy. Scandinavian Journal of Gastroenterology.

[5] Ohkusa T (2001). Improvement in atrophic gastritis and intestinal metaplasia in patients in whom helicobacter pylori was eradicated. Annals of Internal Medicine.

[6] Rakic S, Pesko P, Milicevic M, Gerzic Z (1992). Atrophic chronic gastritis and esophagogastric anastomotic leak after resection and reconstruction for esophageal carcinoma. Journal of Surgical Oncology.

[7] Donzelli A (2016). Helicobacter pylori Eradication?. Gastroenterology.

[8] Tian J (2015). Investigation on the antidepressant effect of sea buckthorn seed oil through the GC-MS-based metabolomics approach coupled with multivariate analysis. Food & function.

[9] Ma Y (2016). Publication Trends in Acupuncture Research: A 20-Year Bibliometric Analysis Based on PubMed. PLoS ONE.

[10] Shu Q (2016). Acupuncture and Moxibustion have Different Effects on Fatigue by Regulating the Autonomic Nervous System: A Pilot Controlled Clinical Trial. Scientific reports.

[11] Cabýoglu MT, Ergene N, Tan U (2006). International Journal of Neuroscience.

[12] Yang M (2017). Moxibustion for pain relief in patients with primary dysmenorrhea: A randomized controlled trial. PLoS ONE.

[13] Bao C (2016). Different brain responses to electro-acupuncture and moxibustion treatment in patients with Crohn’s disease. Scientific reports.

[14] Gu W, Hu QC (2009). Clinical observation on acupuncture for treatment of chronic atrophic gastritis. Chinese acupuncture & moxibustion..

[15] Gao X, Yuan J, Li H, Ren S (2007). Clinical research on acupuncture and moxibustion treatment of chronic atrophic gastritis. Journal of traditional Chinese medicine.

[16] Zhang J (2016). Acupuncture Decreases NF-κB p65, miR-155, and miR-21 and Increases miR-146a Expression in Chronic Atrophic Gastritis Rats. Evidence-Based Complementary and Alternative Medicine.

[17] Yang Z (2015). Effects of moxibustion on cell proliferative factors in gastric mucosa in rats with precancerous lesions of chronic atrophic gastritis. Chinese acupuncture & moxibustion.

[18] Nicholson JK, Lindon JC (2008). Systems biology: Metabonomics. Nature.

[19] Lindon, J. C., Nicholson, J. K. & Holmes, E. *The handbook of metabonomics and metabolomics* (Elsevier, 2011).

[20] Wang X (2011). Potential role of metabolomics apporoaches in the area of traditional Chinese medicine: As pillars of the bridge between Chinese and Western medicine. Journal of Pharmaceutical and Biomedical Analysis.

[21] Li ZY, He P, Sun HF, Qin XM, Du GH (2014). ^1^H NMR based metabolomic study of the antifatigue effect of Astragali Radix. Molecular BioSystems.

[22] Zhang L (2014). Metabonomic Analysis Reveals Efficient Ameliorating Effects of Acupoint Stimulations on the Menopause-caused Alterations in Mammalian Metabolism. Scientific reports.

[23] Ju L (2016). Metabonomic study of the effects of different acupuncture directions on therapeutic efficacy. Journal of Chromatography B.

[24] Xu J (2015). ^1^H NMR Metabolic Profiling of Biofluids from Rats with Gastric Mucosal Lesion and Electroacupuncture Treatment. Evidence-based Complementary and Alternative Medicine: eCAM.

[25] Xu J (2017). NMR-based metabolomics Reveals Alterations of Electro-acupuncture Stimulations on Chronic Atrophic Gastritis Rats. Scientific reports.

[26] Huang W (2013). Effect of amitriptyline on gastrointestinal function and brain-gut peptides: A double-blind trial. World Journal of Gastroenterology: WJG.

[27] Erin N, Türker S, Elpek Ö, Yıldırım B (2012). Differential changes in Substance P, VIP as well as neprilysin levels in patients with gastritis or ulcer. Peptides.

[28] Choi YJ (2016). Increase in plasma acyl ghrelin levels is associated with abatement of dyspepsia following Helicobacter pylori eradication. Journal of gastroenterology.

[29] Tian J (2014). Dynamic analysis of the endogenous metabolites in depressed patients treated with TCM formula Xiaoyaosan using urinary ^1^H NMR-based metabolomics. Journal of Ethnopharm- acology.

[30] El Azzouny M (2016). Knockdown of ATP citrate lyase in pancreatic beta cells does not inhibit insulin secretion or glucose flux and implicates the acetoacetate pathway in insulin secretion. Molecular Metabolism.

[31] Lever M, Slow S (2010). The clinical significance of betaine, an osmolyte with a key role in methyl group metabolism. Clinical biochemistry.

[32] Alirezaei M, Jelodar G, Niknam P, Ghayemi Z, Nazifi S (2011). Betaine prevents ethanol-induced oxidative stress and reduces total homocysteine in the rat cerebellum. Journal of physiology and biochemistry.

[33] Castellano R (2017). Methionine and cysteine deficiencies altered proliferation rate and time-course differentiation of porcine preadipose cells. Amino acids.

[34] akagi, H. & Ohtsu, I. In *Amino* Acid *Fermentation* (eds Atsushi Yokota & Masato Ikeda) 129–151 (Springer Japan, 2017).

[35] Jiang Y (2016). Enzymatic Production of Glutathione by Bifunctional γ-Glutamylcysteine Synthetase/Glutathione Synthetase Coupled with *In Vitro* Acetate Kinase-Based ATP Generation. Applied biochemistry and biotechnology.

[36] Del Buono D, Mimmo T, Terzano R, Tomasi N, Cesco S (2014). Effect of cadmium on antioxidative enzymes, glutathione content, and glutathionylation in tall fescue. Biologia Plantarum.

[37] Franco R (2007). The central role of glutathione in the pathophysiology of human diseases. Archives of Physiology and Biochemistry.

[38] Sgambato D (2016). Gut-Brain Axis in Gastric Mucosal Damage and Protection. Current neuropharmacology.

[39] Holtmann G, Talley NJ (2014). The stomach–brain axis. Best Practice & Research Clinical Gastroenterology.

[40] Ambati CSR, Yuan F, Abu-Elheiga LA, Zhang Y, Shetty V (2017). Identification and Quantitation of Malonic Acid Biomarkers of In-Born Error Metabolism by Targeted Metabolomics. J. Am. Soc. Mass Spectrom..

[41] Klein M, Swinnen S, Thevelein JM, Nevoigt E (2017). Glycerol metabolism and transport in yeast and fungi: established knowledge and ambiguities. Environmental microbiology.

[42] Seybolt SEJ (2010). Is it time to reassess alpha lipoic acid and niacinamide therapy in schizophrenia?. Medical hypotheses.

[43] Fernstrom JD, Fernstrom MH (2007). Tyrosine, Phenylalanine, and Catecholamine Synthesis and Function in the Brain. The Journal of nutrition.

[44] Oliveros A (2017). Label-Free Neuroproteomics of the Hippocampal-Accumbal Circuit Reveals Deficits in Neurotransmitter and Neuropeptide Signaling in Mice Lacking Ethanol-Sensitive Adenosine Transporter. Journal of Proteome Research.

[45] Behm M, Wahlstedt H, Widmark A, Eriksson M, Öhman M (2017). Accumulation of nuclear ADAR2 regulates adenosine-to-inosine RNA editing during neuronal development. Journal of Cell Science.

[46] Beal MW (1999). Acupuncture and Acupressure. Journal of Nurse-Midwifery.

[47] Takahashi T (2011). Mechanism of Acupuncture on Neuromodulation in the Gut—A Review. Neuromodulation:Technology at the Neural Interface.

[48] Liu C-C (2015). Plasma-metabolite-biomarkers for the therapeutic response in depressed patients by the traditional Chinese medicine formula Xiaoyaosan: A ^1^H NMR-based metabolomics approach. Journal of Affective Disorders.

