# Author Correction: Comparative metabolomics study on therapeutic mechanism of electro-acupuncture and moxibustion on rats with chronic atrophic gastritis (CAG)

**DOI:** 10.1038/s41598-022-07361-7

**Published:** 2022-02-28

**Authors:** Cai-chun Liu, Jiao-long Chen, Xiao-rong Chang, Qi-da He, Jia-cheng Shen, Lin-yu Lian, Ya-dong Wang, Yuan Zhang, Fu-qiang Ma, Hui-ying Huang, Zong-bao Yang

**Affiliations:** 1 Department of Traditional Chinese Medicine and Shenzhen Research Institute, Xiamen University, Xiamen, 361005 China; 2 College of Acupuncture and Moxibustion, Fujian University of Traditional Chinese Medicine, Fuzhou, 350122 China; 3 College of Acupuncture and Moxibustion, Hunan University of Traditional Chinese Medicine, Changsha, 410208 China; 4 School of Life Sciences, Xiamen University, Xiamen, 361005 China

Correction to: *Scientific Reports* https://doi.org/10.1038/s41598-017-13195-5, published online 30 October 2017

This Article contains an error in the legend of Figure 1.

“Histological examination of gastric mucosa from six groups. (**A** and a, the control rats; **B** and b, the CAG rats; **C** and c, CAG rats with electro-acupuncture treatment on the stomach meridian acupoints; **D** and d, CAG rats with electro-acupuncture treatment on the stomach meridian acupoints; **E** and e, CAG rats with moxibustion treatment on the stomach meridian acupoints; **F** and f, CAG rats with moxibustion on non-acupoints). Scale bars represent 2 μm for the top row and 0.5 μm for the bottom row.”

should read:

“Histological examination of gastric mucosa from six groups. (**A** and a, the control rats; **B** and b, the CAG rats; **C** and c, CAG rats with electro-acupuncture treatment on the stomach meridian acupoints; **D** and d, CAG rats with electro-acupuncture treatment on the stomach meridian non-acupoints; **E** and e, CAG rats with moxibustion treatment on the stomach meridian acupoints; **F** and f, CAG rats with moxibustion on non-acupoints). Scale bars represent 2 μm for the top row and 0.5 μm for the bottom row.”

