# Supplementary material for: Comparative metabolomics study on therapeutic mechanism of electro-acupuncture and moxibustion on rats with chronic atrophic gastritis (CAG)
Source: Sci Rep. 2017 Oct 30;7:14362. doi: 10.1038/s41598-017-13195-5 (PMC5662682; doi:10.1038/s41598-017-13195-5)
Supplement: Supplementary file 1 — Supplementary Information [file 41598_2017_13195_MOESM1_ESM.doc]

**Comparative metabolomics study on therapeutic mechanism of electro-acupuncture and moxibustion on rats with chronic atrophic gastritis（CAG）**

Cai-chun Liu1, Jiao-long Chen†1,2, Xiao-rong Chang3, Qi-da He1, 2, Jia-cheng Shen1, Lin-yu Lian1,2, Ya-dong Wang1, Yuan Zhang1,3, Fu-qiang Ma1, Hui-ying Huang4, Zong-bao Yang1,*

1Department of Traditional Chinese Medicine and Shenzhen Research Institute, Xiamen University, Xiamen 361005, China

2 College of Acupuncture and Moxibustion, Fujian university of Traditional Chinese Medicine, Fuzhou 350122, China

3 College of Acupuncture and Moxibustion, Hunan university of Traditional Chinese Medicine, Changsha 410208, China

4 School of Life Sciences, Xiamen University, Xiamen 361005, China

*Correspondence and requests for materials should be addressed to Zong-bao Yang（[yangzb@xmu.edu.cn](mailto:yangzb@xmu.edu.cn)）


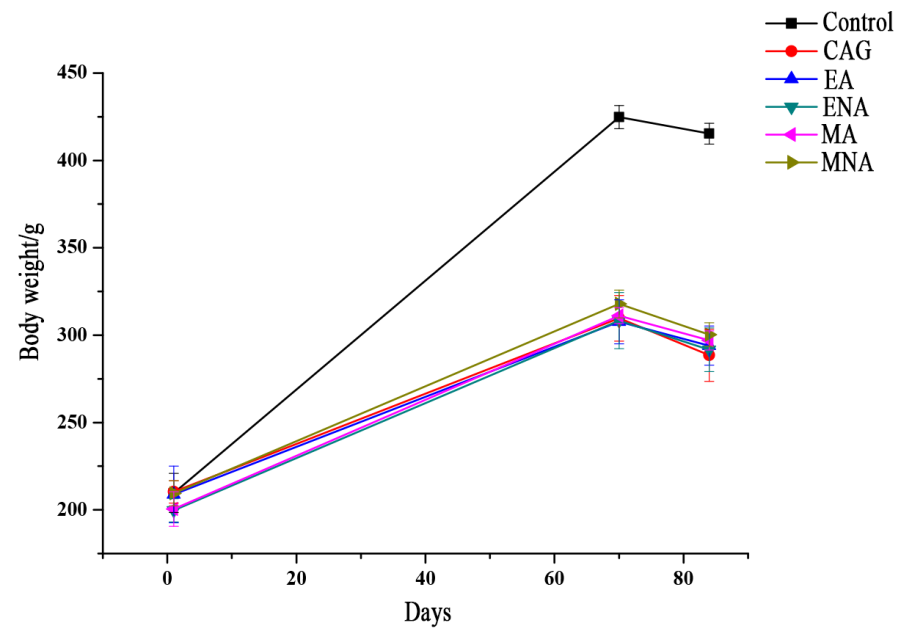


**Fig.S1** Plot of weight trend in all groups. Values are expressed as mean ± SD (n=6). (control, the control group; CAG, chronic atrophic gastritis group; EA, CAG rats with electro-acupuncture treatment on the stomach meridianacupoints; ENA, CAG rats with electro-acupuncture on non-acupoints; MA, CAG rats with moxibustion treatment on the stomach meridian acupoints; MNA, CAG rats with moxibustion on non-acupoints).


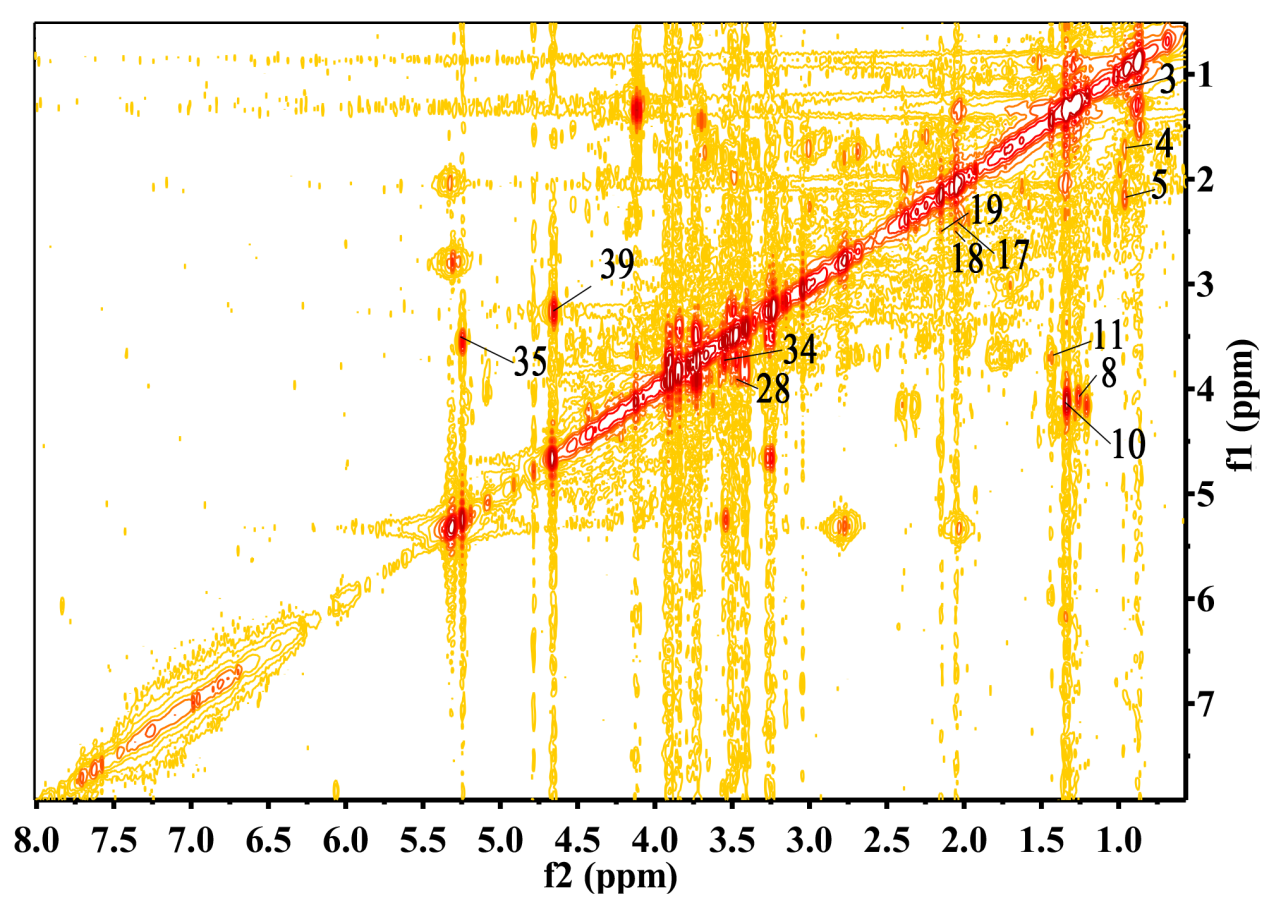


**Fig.S2.** Portion of the 1H−1H COSY 600 MHz spectrum of serum metabolites. Identified metabolites: 3, Isoleucine; 4, Leucine; 5, Valine; 8, β-OH-butyrate; 10, Lactate; 11, Alanine; 17, Glutamine; 18, Methionine; 19, Glutathione; 28, Choline; 34, Glycerol; 35, Glycogen; 39, Glucose.


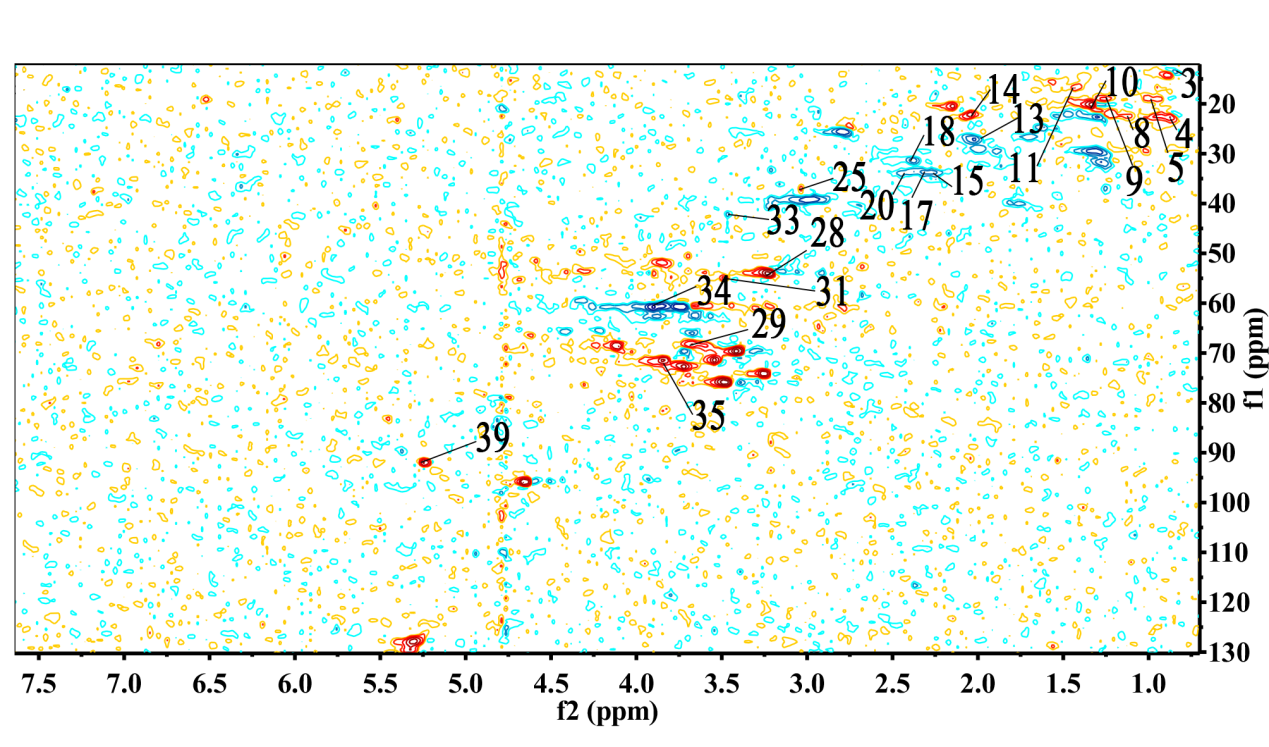


**Fig.S3.** Portion of the1H−13C HSQC 600 MHz spectrum of serum metabolites. Identified metabolites: 3, Isoleucine; 4, Leucine; 5, Valine; 8, β-OH-butyrate; 9, Methylmalonate; 10, Lactate; 11, Alanine; 13, Acetate; 14, N-acetyl aspartate;15, Glutamate; 17, Glutamine; 18, Methionine; 20, Acetone; 25, Creatinine; 28, Choline; 29, Phosphocholine; 31, Betaine; 33, Glycine; 34, Glycerol; 35, Glycogen; 39, Glucose.

**Table S1** Peak attribution of the main marked metabolites in 1H-NMR spectra of serum sample

| **NO.** | **Metabolites** | **δ1H/ppm** | **Moieties** | **δ13C/ppm** | **Assigned with** |
| --- | --- | --- | --- | --- | --- |
| 1 | LDL | 0.86(br) | CH3-(CH2)n- |  |  |
| 2 | VLDL | 0.89(br) | CH3-(CH2)n- |  |  |
| 3 | Isoleucine | 0.94(t); 1.01(d) | δ-CH3; β-CH3 | 13.85 | COSY,HSQC |
| 4 | Leucine | 0.96(t); 1.70(m) | CH3; CH2&γ-CH | 23.57 | COSY,HSQC |
| 5 | Valine | 0.99(d); 1.04(d) | γ-CH3; γ-CH´3 | 19.42 | COSY,HSQC |
| 6 | 2-Ketobutyric acid | 1.07(t) | CH3 |  |  |
| 7 | Ethanol | 1.19(t) | CH3 |  |  |
| 8 | β-OH-butyrate | 1.20(d) | γCH3 | 22.78 | COSY,HSQC |
| 9 | Methylmalonate | 1.23(d) | CH3 | 17.92 | HSQC |
| 10 | Lactate | 1.33(d); 4.11(q) | CH3; CH | 22.90 | COSY,HSQC |
| 11 | Alanine | 1.48(d); 3.78(q) | CH3; CH | 18.03 | COSY,HSQC |
| 13 | Acetate | 1.92(s) | CH3 | 26.92 | HSQC |
| 15 | Glutamate | 2.05(m) | β-CH | 32.87 | HSQC |
| 16 | O-acetyl glycoprotein | 2.08(s) | CH3 |  | HSQC |
| 17 | Glutamine | 2.14(m) | β-CH2 | 32.79 | COSY,HSQC |
| 18 | Methionine | 2.14(s) | -CH3 | 32.72 | COSY,HSQC |
| 19 | Glutathione | 2.17(m); 2.55(m) | β-CH2; γ-CH2 | 34.12 | COSY |
| 20 | Acetone | 2.23(s);2,45 | CH3 |  | HSQC |
| 21 | Acetoacetate | 2.30(s); 3.45(s) | CH3; CH2 | 33.12 |  |
| 22 | Citrate | 2.54(d) | CH2 |  |  |
| 25 | Creatinine | 3.05(s); 4.06(s) | CH3; CH2 | 34.92 | HSQC |
| 26 | Phenylalanine | 3.28(m);7.33(d); 7.38(t) | β-CH´; o-CH; p-CH |  |  |
| 28 | Choline | 3.20(s);3.52(m);4.07(m) | CH3;N-CH2;O-CH2 | 54.92 | HSQC |
| 29 | Phosphocholine | 3.22(s);3.59(m);4.17(m) | CH3;N-CH2;O-CH2 | 68.91 | HSQC |
| 30 | Glycerophosphocholine | 3.23(s); 3.96(m) | CH3; CH&O-CH2 |  |  |
| 31 | Betaine | 3.27(s); 3.89(s) | CH3; CH2 | 55.12 | HSQC |
| 33 | Glycine | 3.56(s) | CH2 | 43.12 | HSQC |
| 34 | Glycerol | 3.57(m);3.62(m);3.79(m) | CH2; CH'2; CH | 65.13 | COSY,HSQC |
| 35 | Glycogen | 3.63(m); 5.40(m) | CH(3,5,29);CH(2) | 72.92 | COSY,HSQC |
| 39 | Glucose | 4.63(d); 5.23(d); | CH(2) | 92.4 | COSY,HSQC |
| 49 | Tyrosine | 6.89(d); 7.19(d) | m-CH; o-CH |  |  |
| 50 | Histidine | 3.14(m); 7.08(s);7.90(d) | β-CH;CH(5);CH(2); |  |  |
| 51 | Methylhistidine | 7.06(s); 7.78(s) | CH(2); CH(4) |  |  |
| 52 | Formate | 8.46(s) | CH |  |  |

a s: singlet, d: doublet, t: triplet, q: quartet, m: multiplet, dd: doublet of doublet, br: broad..

b LDL: Low density lipoprotein; VLDL: Very low density lipoprotein.


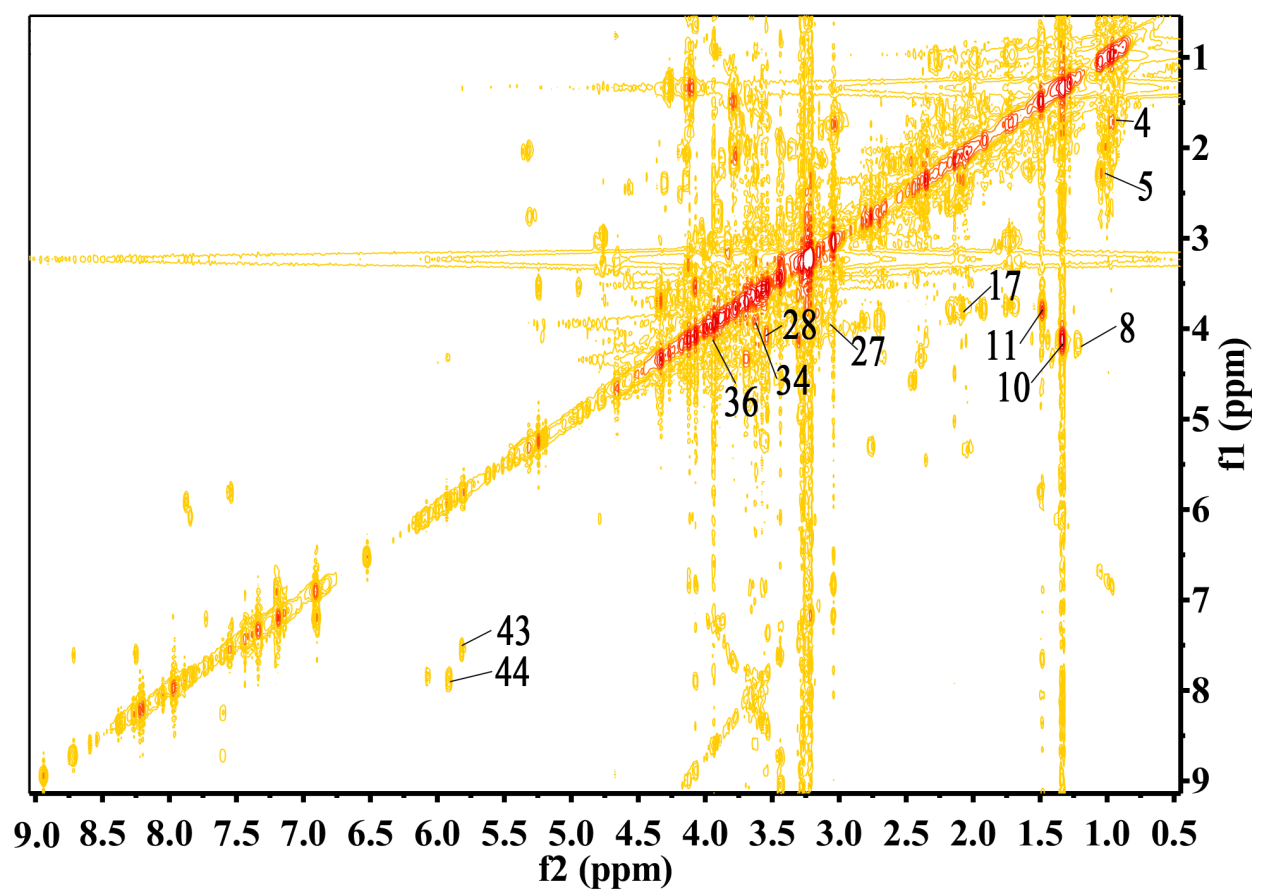


**Fig.S4.** Portion of the 1H−1H COSY 600 MHz spectrum of stomach tissues metabolites. Identified metabolites: 4, Leucine; 5, Valine; 8, β-OH-butyrate; 10, Lactate; 11, Alanine; 17, Glutamine; 27, Ethanolamine; 28, Choline; 34, Glycerol; 36, Serine; 43, Uracil; 44, Uridine.


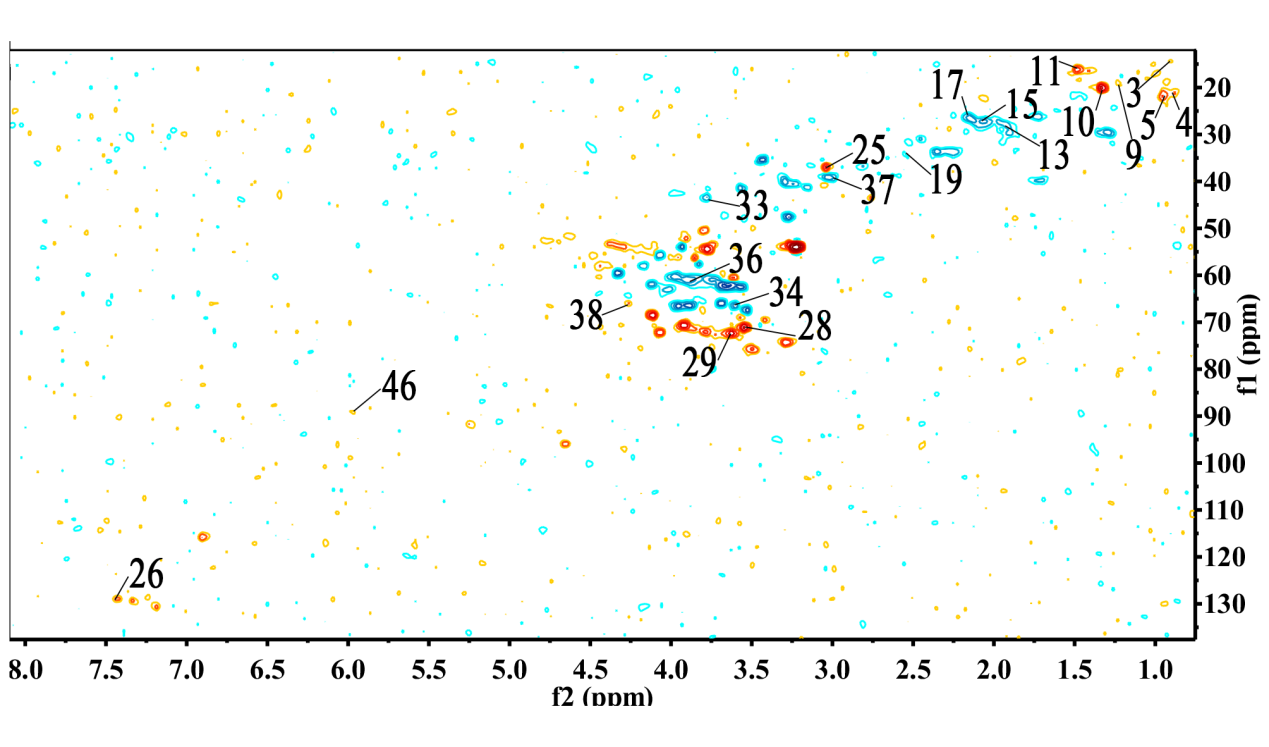


**Fig.S5.** Portion of the 1H−1H COSY 600 MHz spectrum of stomach tissues metabolites. Identified metabolites: 3, Isoleucine; 4, Leucine; 5, Valine; 9, Methylmalonate; 10, Lactate; 11, Alanine; 13, Acetate; 15, Glutamate; 17, Glutamine; 19, Glutathione; 25, Creatinine; 26, Phenylalanine; 28, Choline; 29, Phosphocholine; 33, Glycine; 34, Glycerol; 36, Serine; 38, Adenosine monophosphate; 46, NADP+.

**Table S2** Peak attribution of the main marked metabolites in 1H-NMR spectra of stomach sample

| **NO.** | **Metabolites** | **δ1H/ppm** | **Moieties** | **δ13C/ppm** | **Assigned with** |
| --- | --- | --- | --- | --- | --- |
| 3 | Isoleucine | 0.94(t); 1.01(d) | δ-CH3; β-CH3 | 14.13 | HSQC |
| 4 | Leucine | 0.96(t); 1.70(m) | CH3; CH2&γ-CH | 22.13 | COSY,HSQC |
| 5 | Valine | 0.99(d); 1.04(d) | γ-CH3; γ-CH´3 | 21.17 | COSY,HSQC |
| 8 | β-OH-butyrate | 1.20(d) | γCH3 |  | COSY |
| 9 | Methylmalonate | 1.23(d) | CH3 | 17.92 | HSQC |
| 10 | Lactate | 1.33(d); 4.11(q) | CH3; CH | 21.85 | COSY,HSQC |
| 11 | Alanine | 1.48(d); 3.78(q) | CH3; CH | 17.52 | COSY,HSQC |
| 13 | Acetate | 1.92(s) | CH3 | 27.4 | HSQC |
| 15 | Glutamate | 2.05(m) | β-CH | 30.78 | HSQC |
| 17 | Glutamine | 2.14(m) | β-CH2 | 27.87 | COSY,HSQC |
| 19 | Glutathione | 2.17(m); 2.55(m) | β-CH2; γ-CH2 | 34.12 | HSQC |
| 25 | Creatinine | 3.05(s) | CH3 | 35.83 | HSQC |
| 26 | Phenylalanine | ;7.33(d); 7.38(t) | β-CH´; o-CH; p-CH | 129.3 | HSQC |
| 27 | Ethanolamine | 3.15(t) 3.84(t) | CH2NH2; CH2OH | 41.36 | COSY,HSQC |
| 28 | Choline | 3.20(s);3.52(m);4.07(m) | CH3;N-CH2;O-CH2 | 54.92 | COSY,HSQC |
| 29 | Phosphocholine | 3.22(s);3.59(m);4.17(m) | CH3;N-CH2;O-CH2 | 71.83 | HSQC |
| 30 | Glycerophosphocholine | 3.23(s); 3.96(m) | CH3; CH&O-CH2 |  |  |
| 33 | Glycine | 3.56(s) | CH2 | 44.25 | HSQC |
| 34 | Glycerol | 3.57(m);3.62(m);3.79(m) | CH2; CH'2; CH | 65.93 | COSY,HSQC |
| 36 | Serine | 3.83(dd); 3.96(m) | CH; CH2 | 61.9 | COSY,HSQC |
| 37 | **Phosphocreatine** | 3.93(s); | CH2 | 39.21 | HSQC |
| 38 | Adenosine monophosphate | 4.03(m); 4.37(m) | O-CH2; CH; | 66.92 | HSQC |
| 40 | Hypoxanthine | 8.19(s); 8.21(s) | CH(2); CH(7) |  |  |
| 41 | Inosine | 4.28(dd); 8.22(s) | CH(5); N-CH=N |  |  |
| 42 | Allantoin | 5.39(s) | CH |  |  |
| 43 | Uracil | 5.80(d); 7.53(d) | CH(5); CH(6) |  | COSY, |
| 44 | Uridine | 5.90(d); 7.87(d) | CH(10); CH(11) |  | COSY, |
| 45 | UDG | 5.97(m) | CH(21,31) |  |  |
| 46 | NADP+ | 6.05(d);6.15(d) | CH(32); CH(2) | 89.23 | HSQC |
| 47 | Inosinic acid | 6.13(d) | CH(2) |  |  |
| 48 | Fumarate | 6.52(s) | CH |  |  |
| 53 | Adenosine | 4.30(dd);8.26(s); 8.35(s) | CH(5); N-CH=N |  |  |
| 54 | Xanthine | 7.93(s) | CH(2);CH(9) |  |  |
| 56 | Nicotinamide | 8.24(dd);8.72(dd); | CH(4);CH(6);CH(2) |  |  |

a s: singlet, d: doublet, t: triplet, q: quartet, m: multiplet, dd: doublet of doublet,

b UDG: Uridine diphosphate glucose


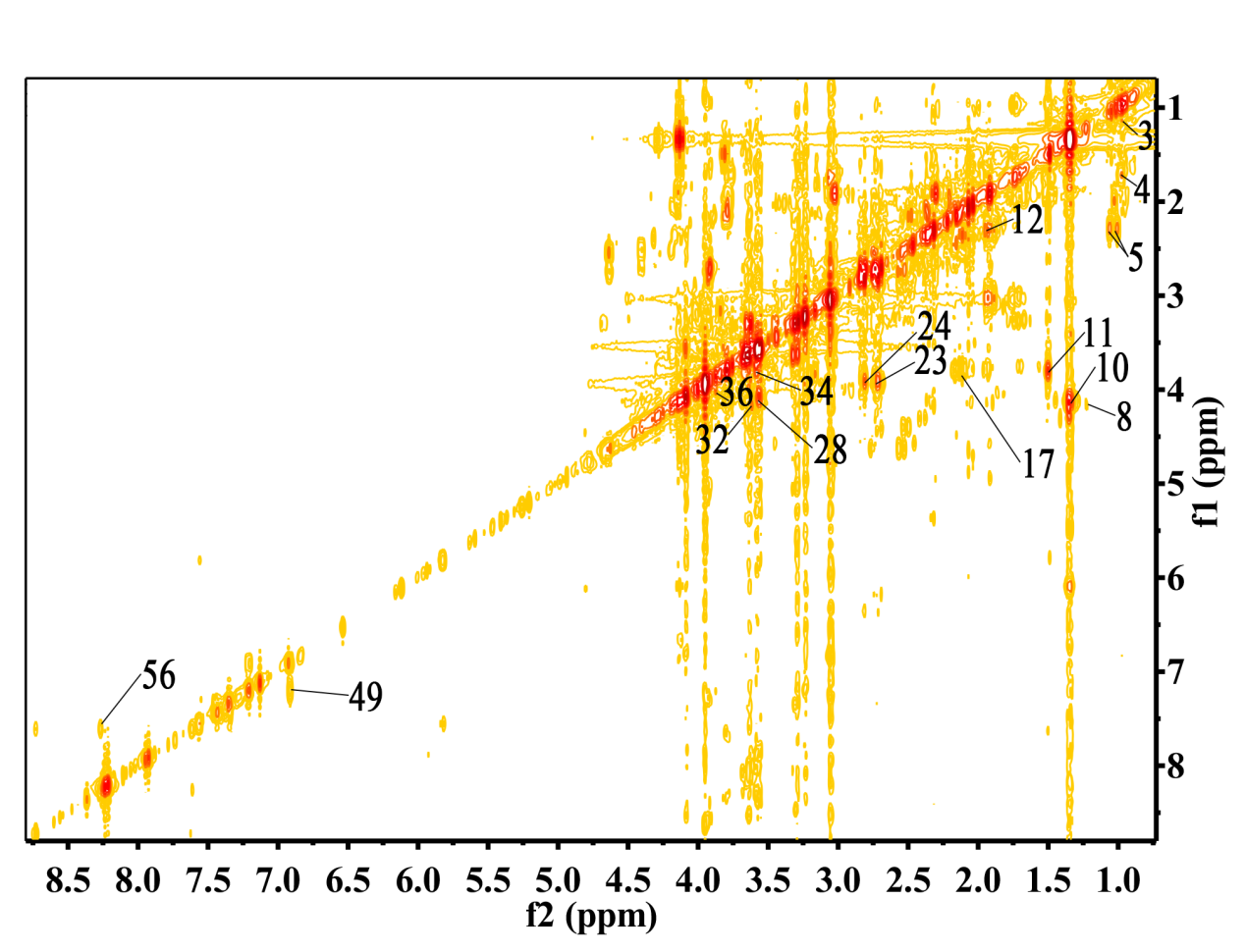


**Fig.S6.** Portion of the 1H−1H COSY 600 MHz spectrum of medulla tissues metabolites. Identified metabolites: 3, Isoleucine; 4, Leucine; 5, Valine; 8, β-OH-butyrate; 10, Lactate; 11, Alanine; 12, γ-Aminobutyrate; 17, Glutamine; 23, Aspartate; 24, N,N-dimethylglycine; 28, Choline; 32, Inositol; 34, Glycerol; 36, Serine; 49, Tyrosine; 56, Nicotinamide.


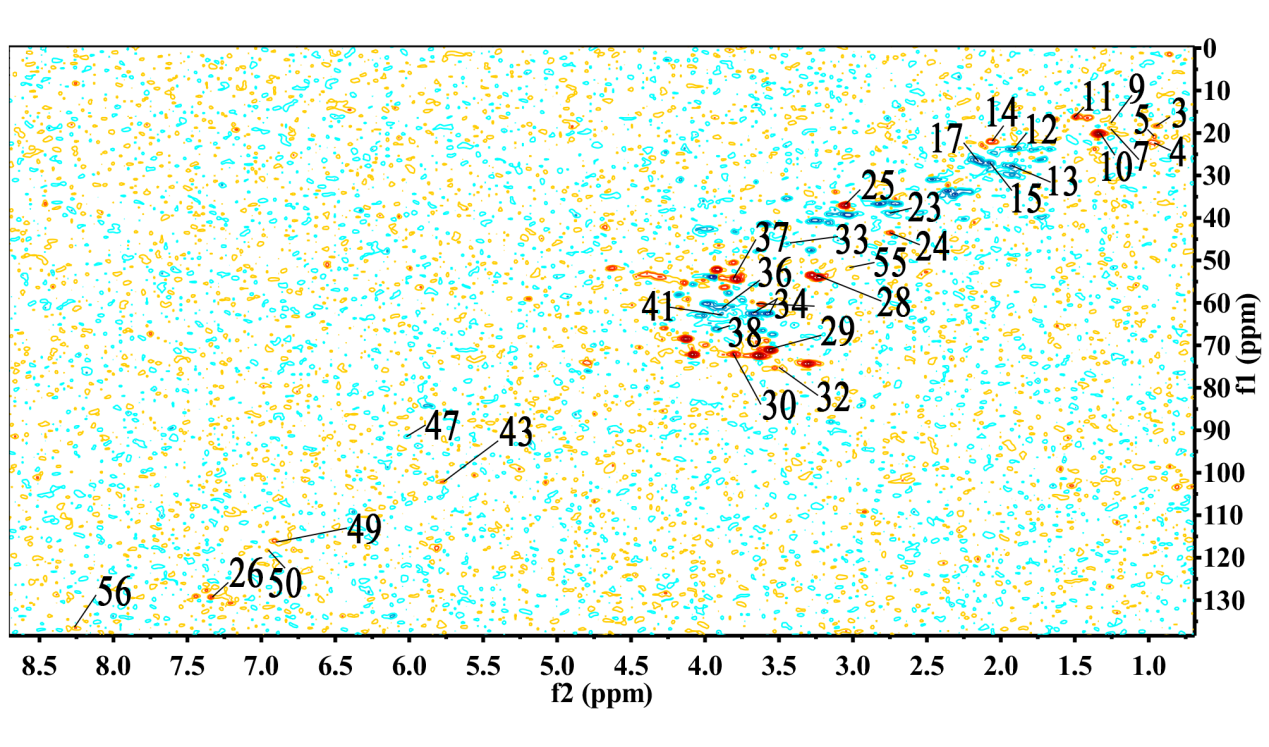


**Fig.S7.** Portion of the 1H−1H COSY 600 MHz spectrum of medulla tissues metabolites. Identified metabolites: 3, Isoleucine; 4, Leucine; 5, Valine; 7, Ethanol; 9, Methylmalonate; 10, Lactate; 11, Alanine; 12, γ-Aminobutyrate; 13, Acetate; 14, N-acetyl aspartate;15, Glutamate; 17, Glutamine; 23, Aspartate; 24, N,N-dimethylglycine; 25, Creatinine; 26, Phenylalanine; 28, Choline; 29, Phosphocholine; 30, Glycerophosphocholine; 32, Inositol; 33, Glycine; 34, Glycerol; 36, Serine; 37,Phosphocreatine**; 38,** Adenosine monophosphate; 41, Inosine; 43, Uracil; 47, Inosinic acid; 49, Tyrosine; 50, Histidine; 55, Malonic acid; 56, Nicotinamide.

**Table S3** Peak attribution of the main marked metabolites in 1H-NMR spectra of medulla sample

| **NO.** | **Metabolites** | **δ1H/ppm** | **Moieties** | **δ13C/ppm** | **Assigned with** |
| --- | --- | --- | --- | --- | --- |
| 3 | Isoleucine | 0.94(t); 1.01(d) | δ-CH3; β-CH3 | 17.75 | COSY,HSQC |
| 4 | Leucine | 0.96(t); 1.70(m) | CH3; CH2&γ-CH | 22.41 | COSY,HSQC |
| 5 | Valine | 0.99(d); 1.04(d) | γ-CH3; γ-CH´3 | 20.76 | COSY,HSQC |
| 7 | Ethanol | 1.19(t) | CH3 | 19.21 | HSQC |
| 8 | β-OH-butyrate | 1.20(d) | γCH3 |  | COSY |
| 9 | Methylmalonate | 1.23(d) | CH3 | 17.83 | HSQC |
| 10 | Lactate | 1.33(d); 4.11(q) | CH3; CH | 20.62 | COSY,HSQC |
| 11 | Alanine | 1.48(d); 3.78(q) | CH3; CH | 16.43 | COSY,HSQC |
| 12 | γ-Aminobutyrate | 1.90(m);2.30(t); | α-CH2;β-CH2 | 24.55 | COSY,HSQC |
| 13 | Acetate | 1.92(s) | CH3 | 27.54 | HSQC |
| 14 | N-acetyl aspartate | 2.02(s) | CH3 | 22.67 | HSQC |
| 15 | Glutamate | 2.05(m) | β-CH | 27.80 | HSQC |
| 17 | Glutamine | 2.14(m) | β-CH2 | 27.33 | COSY,HSQC |
| 23 | Aspartate | 2.67(dd);2.82(dd); | β-CH; β-CH' | 39.33 | COSY,HSQC |
| 24 | N,N-dimethylglycine | 2.93(s); 3.73(s) | CH3; CH2 | 43.93 | ,HSQC |
| 25 | Creatinine | 3.05(s); 4.06(s) | CH3; CH2 | 36.21 | HSQC |
| 26 | Phenylalanine | 7.33(d); 7.38(t) | β-CH´; o-CH; p-CH | 130.28 | HSQC |
| 28 | Choline | 3.20(s);3.52(m);4.07(m) | CH3;N-CH2;O-CH2 | 54.92 | COSY;HSQC |
| 29 | Phosphocholine | 3.22(s);3.59(m);4.17(m) | CH3;N-CH2;O-CH2 | 70.46 | HSQC |
| 30 | Glycerophosphocholine | 3.23(s); 3.96(m) | CH3; CH&O-CH2 | 72.01 | HSQC |
| 32 | Inositol | 3.28(t); 3.54(dd) | CH(2); CH(4, 6); | 75.12 | HSQC |
| 33 | Glycine | 3.56(s) | CH2 | 43.12 | HSQC |
| 34 | Glycerol | 3.57(m);3.62(m);3.79(m) | CH2; CH'2; CH | 65.13 | COSY,HSQC |
| 36 | Serine | 3.83(dd); 3.96(m) | CH; CH2 | 60.02 | COSY,HSQC |
| 37 | **Phosphocreatine** | 3.93(s); | CH2 | 54.97 | HSQC |
| 38 | Adenosine monophosphate | 4.03(m); 4.37(m) | O-CH2; CH; | 66.07 | HSQC |
| 40 | Hypoxanthine | 8.19(s); 8.21(s) | CH(2); CH(7) |  |  |
| 41 | Inosine | 4.28(dd); 8.22(s) | CH(5); N-CH=N | 62.7 | HSQC |
| 42 | Allantoin | 5.39(s) | CH |  |  |
| 43 | Uracil | 5.80(d); 7.53(d) | CH(5); CH(6) | 102.24 | HSQC |
| 44 | Uridine | 5.90(d); 7.87(d) | CH(10); CH(11) |  |  |
| 47 | Inosinic acid | 6.13(d) | CH(2) | 90.87 | HSQC |
| 48 | Fumarate | 6.52(s) | CH |  |  |
| 49 | Tyrosine | 6.89(d); 7.19(d) | m-CH; o-CH | 118.72 | COSY;HSQC |
| 50 | Histidine | 7.08(s);7.90(d) | β-CH;CH(5);CH(2); | 120.86 | HSQC |
| 51 | Methylhistidine | 7.06(s); 7.78(s) | CH(2); CH(4) |  |  |
| 52 | Formate | 8.46(s) | CH |  |  |
| 53 | Adenosine | 8.26(s); 8.35(s) | CH(5); N-CH=N |  |  |
| 54 | Xanthine | 7.93(s) | CH(2);CH(9) |  |  |
| 55 | Malonic acid | 3.11(s) | CH2 | 51.23 | HSQC |
| 56 | Nicotinamide | 8.24(dd);8.72(dd); | CH(4);CH(6);CH(2) | 137.46 | COSY; HSQC |

a s: singlet, d: doublet, t: triplet, q: quartet, m: multiplet, dd: doublet of doublet.

c LDL: Low density lipoprotein; VLDL: Very low density lipoprotein;


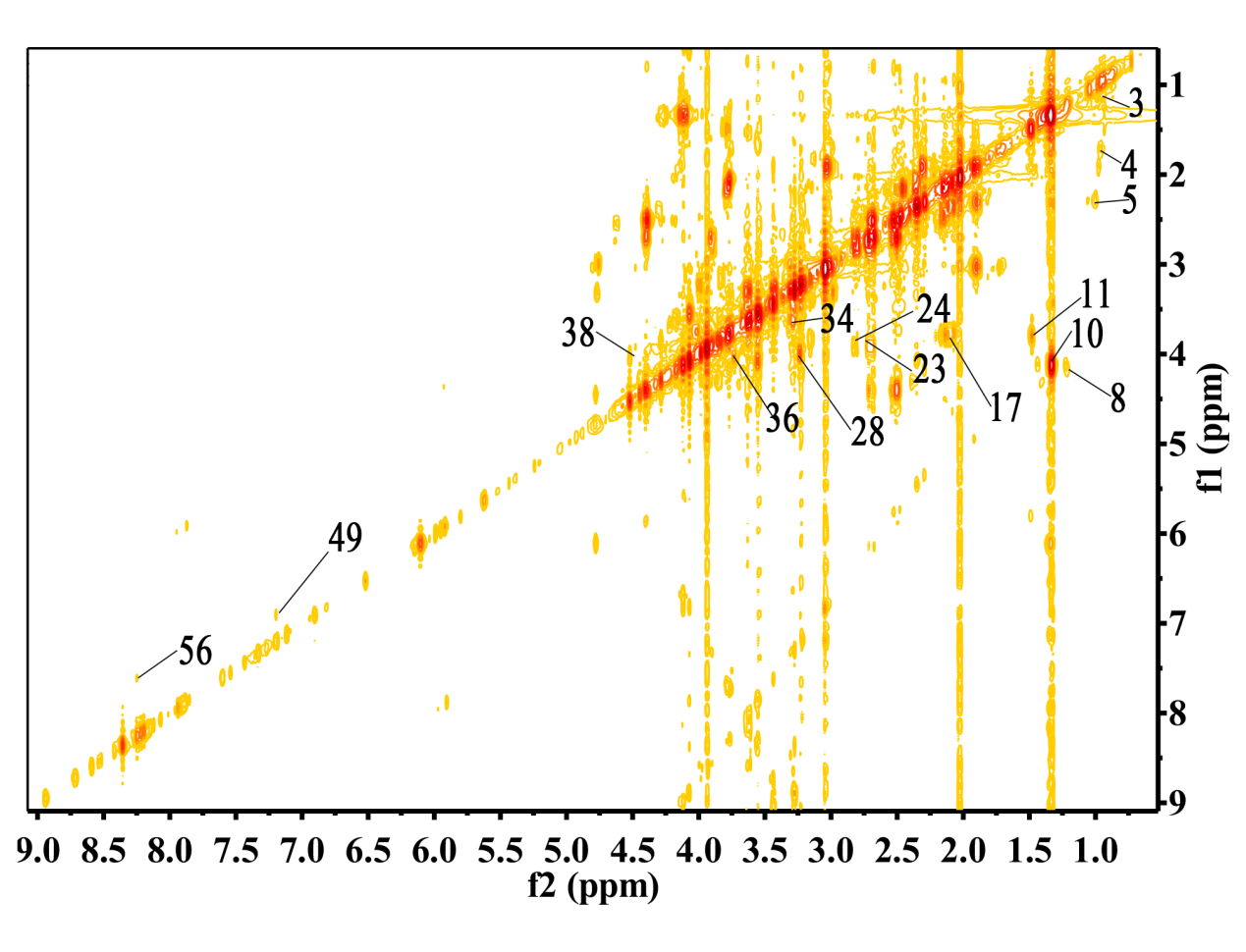


**Fig.S8.** Portion of the 1H−1H COSY 600 MHz spectrum of cerebral cortex tissues metabolites. Identified metabolites: 3, Isoleucine; 4, Leucine; 5, Valine; 8, β-OH-butyrate; 10, Lactate; 11, Alanine; 17, Glutamine; 23, Aspartate; 24, N,N-dimethylglycine; 28, Choline; 36, Serine; **38,** Adenosine monophosphate; 49, Tyrosine; 56, Nicotinamide.


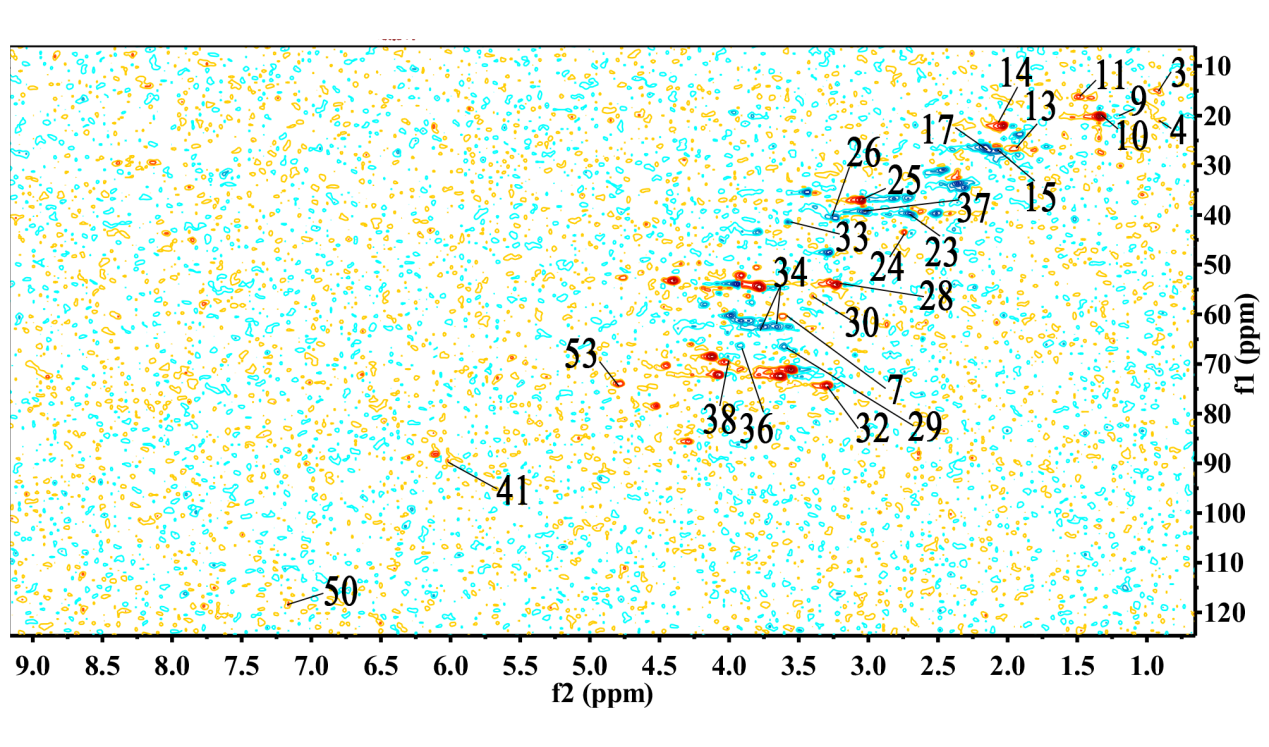


**Fig.S9.** Portion of the 1H−1H COSY 600 MHz spectrum of cerebral cortex tissues metabolites. Identified metabolites: 3, Isoleucine; 4, Leucine; 7, Ethanol; 9, Methylmalonate; 10, Lactate; 11, Alanine; 13, Acetate; 14, N-acetyl aspartate;15, Glutamate; 17, Glutamine; 23, Aspartate; 24, N,N-dimethylglycine; 25, Creatinine; 26, Phenylalanine; 28, Choline; 29, Phosphocholine; 30, Glycerophosphocholine; 32, Inositol; 33, Glycine; 34, Glycerol; 36, Serine; **38,** Adenosine monophosphate; 41, Inosine; 50, Histidine; 53, Adenosine.

**Table S4** Peak attribution of the main marked metabolites in 1H-NMR spectra of cerebral cortex. sample

| **NO.** | **Metabolites** | **δ1H/ppm** | **Moieties** | **δ13C/ppm** | **Assigned with** |
| --- | --- | --- | --- | --- | --- |
| 3 | Isoleucine­­ | 0.94(t); 1.01(d) | δ-CH3; β-CH3 | 14.57 | COSY,HSQC |
| 4 | Leucine | 0.96(t); 1.70(m) | CH3; CH2&γ-CH | 21.57 | COSY,HSQC |
| 5 | Valine | 0.99(d); 1.04(d) | γ-CH3; γ-CH´3 |  | COSY |
| 7 | Ethanol | 1.19(t) | CH3 | 60.02 | HSQC |
| 8 | β-OH-butyrate | 1.20(d) | γCH3 |  | COSY |
| 9 | Methylmalonate | 1.23(d) | CH3 | 19.87 | HSQC |
| 10 | Lactate | 1.33(d); 4.11(q) | CH3; CH | 19.97 | COSY,HSQC |
| 11 | Alanine | 1.48(d); 3.78(q) | CH3; CH | 17.93 | COSY,HSQC |
| 12 | γ-Aminobutyrate | 1.90(m);2.30(t); | α-CH2;β-CH2 | - | - |
| 13 | Acetate | 1.92(s) | CH3 | 25.95 | HSQC |
| 14 | N-acetyl aspartate | 2.02(s) | CH3 | 22.69 | HSQC |
| 15 | Glutamate | 2.05(m) | β-CH | 30.78 | HSQC |
| 17 | Glutamine | 2.14(m) | β-CH2 | 33.91 | COSY,HSQC |
| 23 | Aspartate | 2.67(dd)2.82(dd); | β-CH; β-CH' | 39.86 | COSY,HSQC |
| 24 | N,N-dimethylglycine | 2.93(s); 3.73(s) | CH3; CH2 | 44.37 | COSY,HSQC |
| 25 | Creatinine | 3.05(s); 4.06(s) | CH3; CH2 | 35.98 | HSQC |
| 26 | Phenylalanine | 3.28(m);7.33(d); 7.38(t) | β-CH´; o-CH; p-CH | 40.04 | HSQC |
| 28 | Choline | 3.20(s);3.52(m);4.07(m) | CH3;N-CH2;O-CH2 | 54.92 | COSY,HSQC |
| 29 | Phosphocholine | 3.22(s);3.59(m);4.17(m) | CH3;N-CH2;O-CH2 | 67.83 | HSQC |
| 30 | Glycerophosphocholine | 3.23(s); 3.96(m) | CH3; CH&O-CH2 | 56.06 | HSQC |
| 32 | Inositol | 3.28(t); 3.54(dd) | CH(2); CH(4, 6); | 75.54 | HSQC |
| 33 | Glycine | 3.56(s) | CH2 | 41.95 | HSQC |
| 34 | Glycerol | 3.57(m);3.62(m);3.79(m) | CH2; CH'2; CH | 63.89 | COSY,HSQC |
| 36 | Serine | 3.83(dd); 3.96(m) | CH; CH2 | 65.21 | COSY,HSQC |
| 37 | **Phosphocreatine** | 3.93(s); | CH2 | 39.57 | HSQC |
| 38 | Adenosine monophosphate | 4.03(m); 4.37(m) | O-CH2; CH; | 68.57 | COSY,HSQC |
| 40 | Hypoxanthine | 8.19(s); 8.21(s) | CH(2); CH(7) |  |  |
| 41 | Inosine | 4.28(dd); 8.22(s) | CH(5); N-CH=N | 89.97 | HSQC |
| 43 | Uracil | 5.80(d); 7.53(d) | CH(5); CH(6) |  |  |
| 48 | Fumarate | 6.52(s) | CH |  |  |
| 49 | Tyrosine | 6.89(d); 7.19(d) | m-CH; o-CH |  | COSY |
| 50 | Histidine | 3.14(m); 7.08(s);7.90(d) | β-CH;CH(5);CH(2); | 118.9 | HSQC |
| 51 | Methylhistidine | 7.06(s); 7.78(s) | CH(2); CH(4) |  |  |
| 52 | Formate | 8.46(s) | CH |  |  |
| 53 | Adenosine | 4.30(dd);8.26(s); 8.35(s) | CH(5); N-CH=N | 74.95 | HSQC |
| 54 | Xanthine | 7.93(s) | CH(2);CH(9) |  |  |
| 56 | Nicotinamide | 8.24(dd);8.72(dd); | CH(4);CH(6);CH(2) |  | COSY |

a s: singlet, d: doublet, t: triplet, q: quartet, m: multiplet, dd: doublet of doublet.

**Table S5** *p* (CV-ANOVA) indicating the model quality of OPLS-DA

| *p*(CV-ANOVA) | | | | | |
| --- | --- | --- | --- | --- | --- |
| OPLS-DA model | | serum | stomach | medulla | cerebral cortex |
| CAG vs. | Control | 0.0079 | 0.4483 | 0.2485 | 0.0957 |
|  | EA | 0.0385 | 0.0235 | 0.2911 | 0.5916 |
|  | ENA | 0.0194 | 0.0433 | 0.0456 | 0.0439 |
|  | MA | 0.0046 | 0.0300 | 0.0659 | 0.0475 |
|  | MNA | 0.0015 | 0.0447 | 0.1761 | 0.1761 |

a Grey shadow statistically signiﬁcant models(*p* (CV-ANOVA)<0.05)


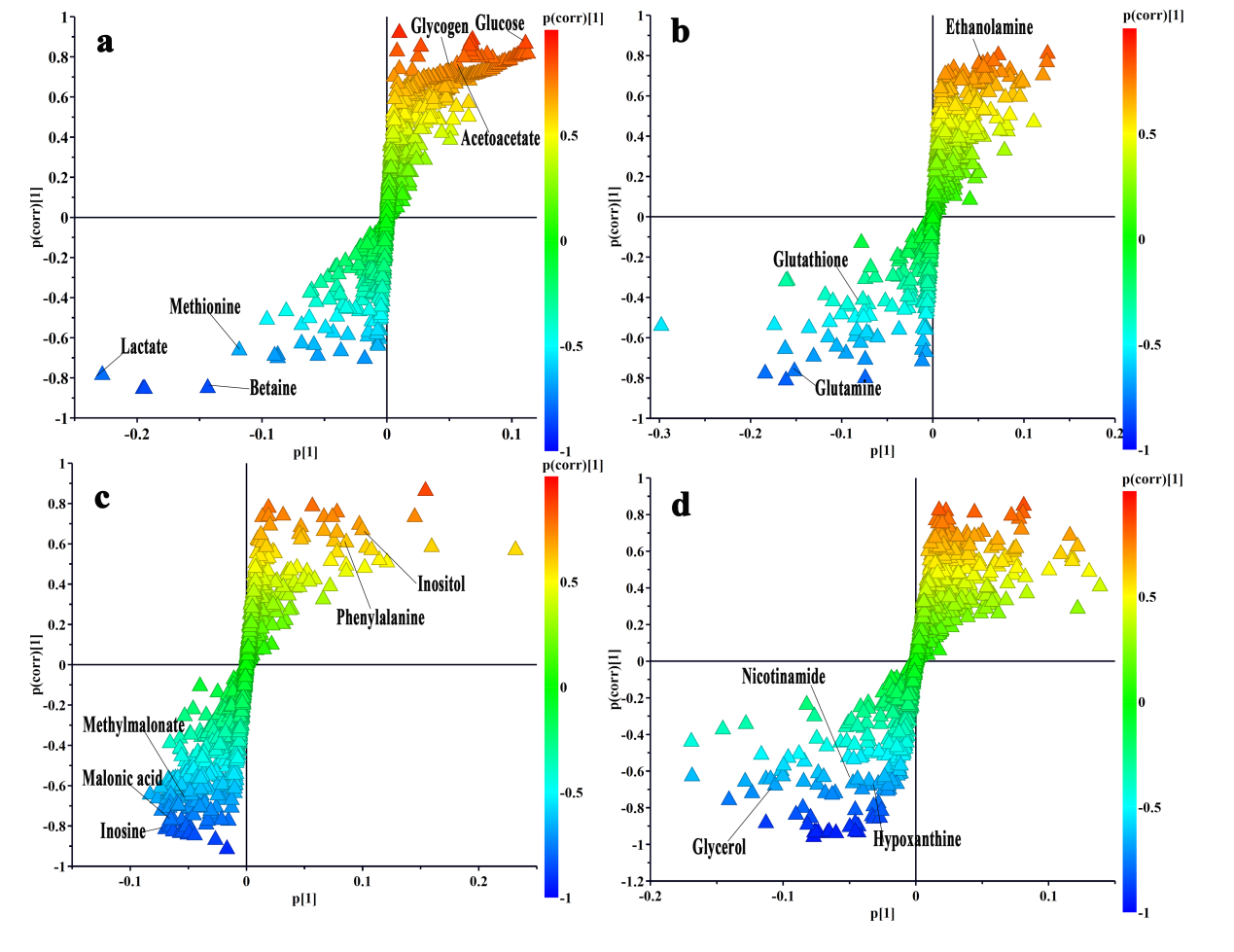


**Fig.S10** the corresponding S-plots of OPLS-DA scores plots from the control group and the CAG group in serum (a), stomach (b), medulla(c) and cerebral cortex (d) tissues.

**
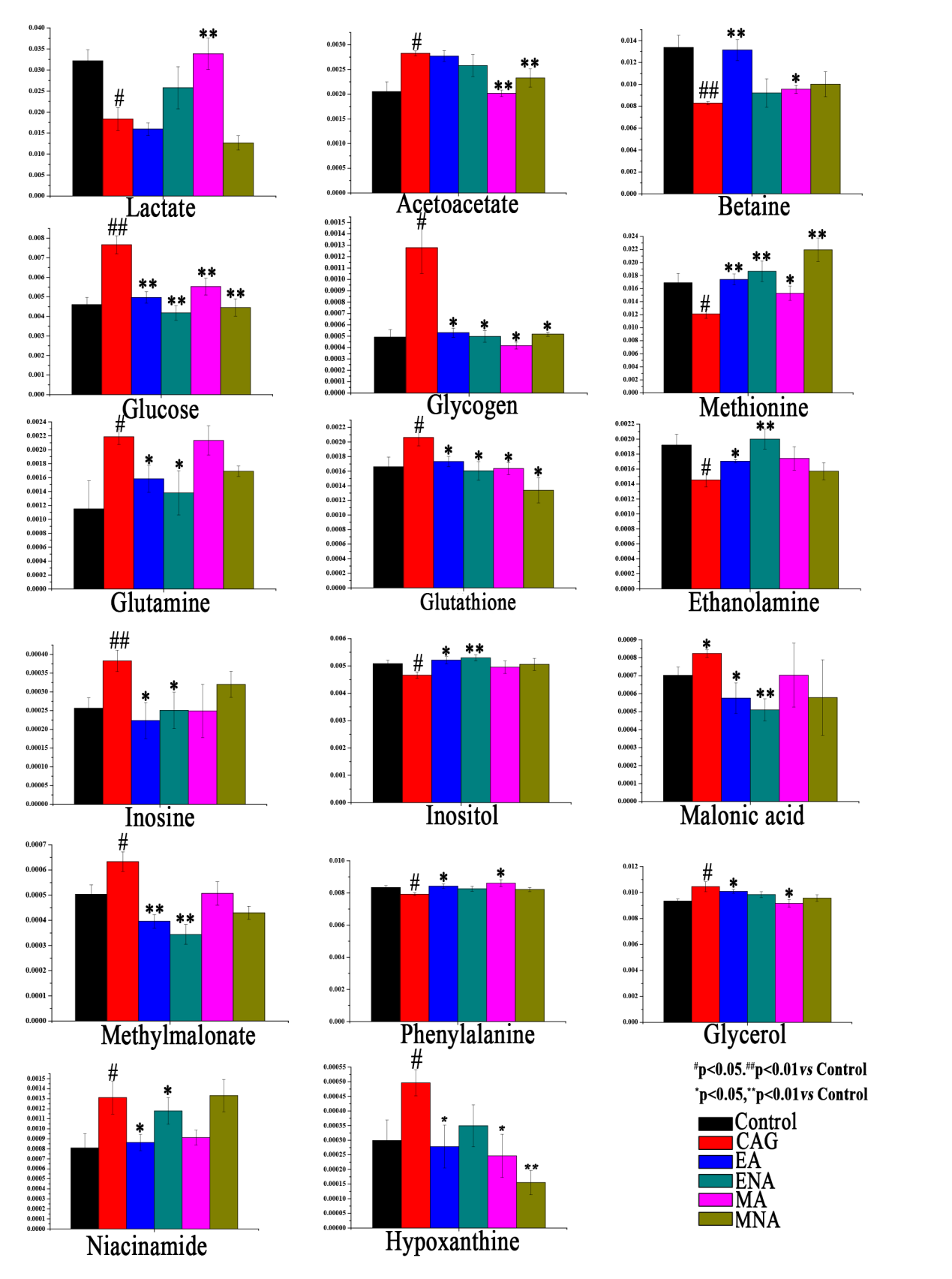
 Fig.S11** Relative abundance (mean ± S.D.) of characteristic metabolites from serum and tissues in six groups (n=6). (Control, control rats; CAG, chronic atrophic gastritis rats; EA, CAG rats with electro-acupuncture treatment on the stomach meridian acupoints; ENA group, CAG rats with electro-acupuncture on non-acupoints; MA group, CAG rats with moxibustion treatment on the stomach meridian acupoints; MNA group, CAG rats with moxibustion on non-acupoints). (# means a statistical significance p < 0.05 when compared with the control group; * means a statistical significance p < 0.05 when compared with the CAG group.)


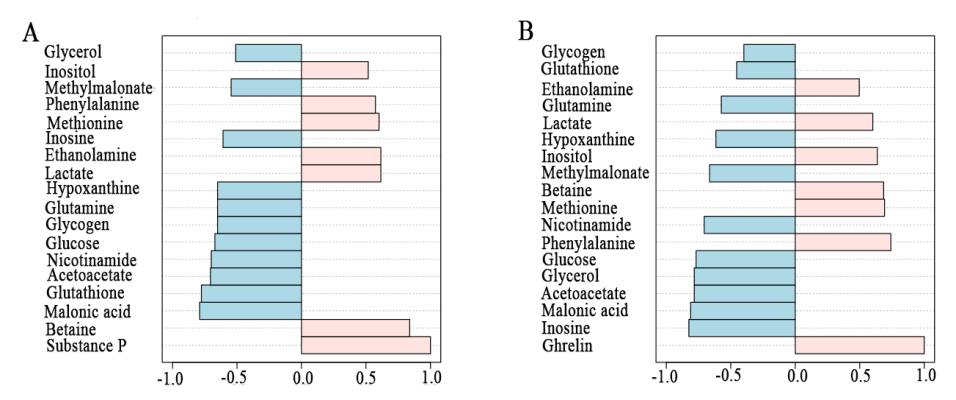


**Fig.S12** Pearson correlation coefﬁcient was presented as bar plots among the potential biomarkers and substance P (A), ghrelin (B). Red bars correspond to positive correlations and blue bars correspond to negative correlations. Bar length reﬂects the magnitude of the correlation coefﬁcients.

*
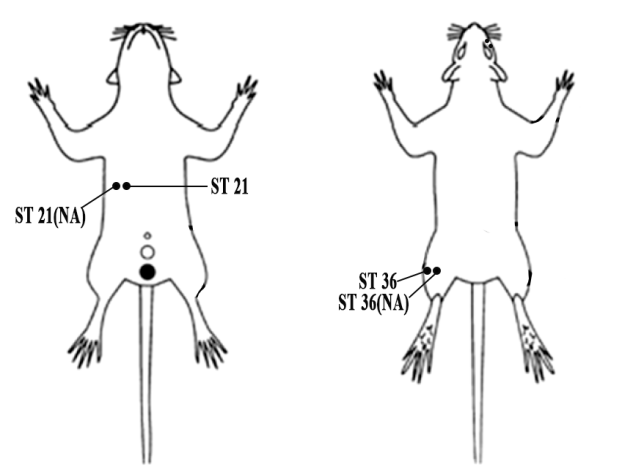
*

**Fig.S13** The selection of acupoints and corresponding non-acupoints for Liangmen (ST 21) and Zusanli (ST 36) on rats.The corresponding non-acupoints located 5 mm away from each of the two acupoints and were entirely unrelated to the stomach meridian and do not lie on any other known acupoints.
